# Stratification of PD-1 blockade response in melanoma using pre- and post-treatment immunophenotyping of peripheral blood

**DOI:** 10.1093/immadv/ltad001

**Published:** 2023-01-06

**Authors:** Natalie M Edner, Elisavet Ntavli, Lina Petersone, Chun Jing Wang, Astrid Fabri, Alexandros Kogimtzis, Vitalijs Ovcinnikovs, Ellen M Ross, Frank Heuts, Yassin Elfaki, Luke P Houghton, Toby Talbot, Amna Sheri, Alexandra Pender, David Chao, Lucy S K Walker

**Affiliations:** Institute of Immunity & Transplantation, University College London Division of Infection & Immunity, London, UK; Institute of Immunity & Transplantation, University College London Division of Infection & Immunity, London, UK; Institute of Immunity & Transplantation, University College London Division of Infection & Immunity, London, UK; Institute of Immunity & Transplantation, University College London Division of Infection & Immunity, London, UK; Institute of Immunity & Transplantation, University College London Division of Infection & Immunity, London, UK; Institute of Immunity & Transplantation, University College London Division of Infection & Immunity, London, UK; Institute of Immunity & Transplantation, University College London Division of Infection & Immunity, London, UK; Institute of Immunity & Transplantation, University College London Division of Infection & Immunity, London, UK; Institute of Immunity & Transplantation, University College London Division of Infection & Immunity, London, UK; Institute of Immunity & Transplantation, University College London Division of Infection & Immunity, London, UK; Institute of Immunity & Transplantation, University College London Division of Infection & Immunity, London, UK; Royal Cornwall Hospitals NHS Trust, Truro, UK; Department of Oncology, Royal Free London NHS Foundation Trust, London, UK; Department of Oncology, Royal Free London NHS Foundation Trust, London, UK; Department of Oncology, Royal Free London NHS Foundation Trust, London, UK; Institute of Immunity & Transplantation, University College London Division of Infection & Immunity, London, UK

**Keywords:** checkpoint inhibition, PD-1 blockade, immunotherapy, biomarker research

## Abstract

Efficacy of checkpoint inhibitor therapies in cancer varies greatly, with some patients showing complete responses while others do not respond and experience progressive disease. We aimed to identify correlates of response and progression following PD-1-directed therapy by immunophenotyping peripheral blood samples from 20 patients with advanced malignant melanoma before and after treatment with the PD-1 blocking antibody pembrolizumab. Our data reveal that individuals responding to PD-1 blockade were characterised by increased CD8 T cell proliferation following treatment, while progression was associated with an increase in CTLA-4-expressing Treg. Remarkably, unsupervised clustering analysis of pre-treatment T cell subsets revealed differences in individuals that went on to respond to PD-1 blockade compared to individuals that did not. These differences mapped to expression of the proliferation marker Ki67 and the costimulatory receptor CD28 as well as the inhibitory molecules 2B4 and KLRG1. While these results require validation in larger patient cohorts, they suggest that flow cytometric analysis of a relatively small number of T cell markers in peripheral blood could potentially allow stratification of PD-1 blockade treatment response prior to therapy initiation.

## Graphical Abstract

**Graphical Abstract:**
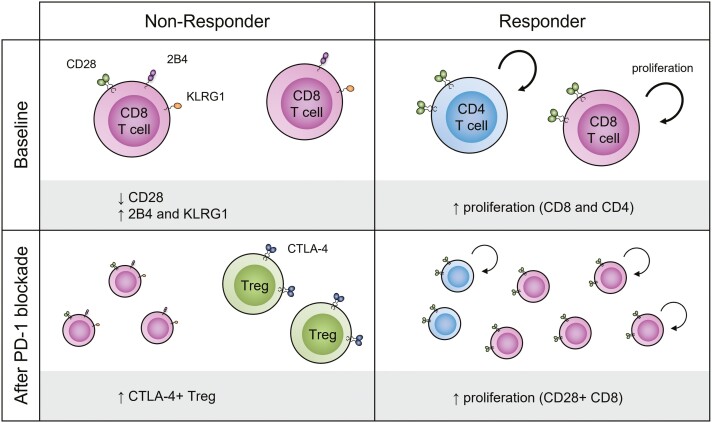

## Introduction

Harnessing the power of the immune system in the form of immunomodulatory drugs has revolutionised cancer therapy, with checkpoint inhibitors targeting the coinhibitory PD-1 and CTLA-4 pathways now being the standard of care in the treatment of many types of malignancies [1]. These therapies aim to reinvigorate tumour-specific T cell responses and release tumour-infiltrating lymphocytes (TILs) from tumour suppression. In particular, antibodies blocking PD-1 or its ligand PD-L1 have caused a paradigm shift in cancer care with an unprecedented 5-year overall survival of 34% for patients with advanced melanoma treated with the PD-1 inhibitor pembrolizumab [2].

Nevertheless, the clinical response following PD-1 pathway interventions shows great heterogeneity and varies across individual patients and different types of tumours [3]. Therefore, considerable effort has been directed towards identification of biomarkers that stratify patient response following therapy. For example, expression of PD-L1 on cells residing within the tumour has been shown to have some positive correlation with response, although patients with PD-L1-negative tumours can still respond to PD-1 blockade [4, 5]. Furthermore, a higher tumour mutational burden and abundance of neoantigens expressed by tumour cells is favourable since it can facilitate anti-tumour immune responses [6, 7]. Similarly, the presence of TILs, particularly CD8 T cells, is associated with a better response following PD-1 blockade [8, 9].

However, to predict patient response using these markers, tumour biopsies, ideally taken at multiple sites, are required, which is not always feasible, and robust biomarkers therefore still present an unmet clinical need in cancer immunotherapy. As an alternative, peripheral blood can provide a snapshot of the systemic immune response and is easily accessible. Tumour-specific T cells have been detected in the circulation and T cell clones reinvigorated by PD-1 blockade can be found in the blood as well as tumour infiltrates [10–12]. Relatively few studies have investigated potential biomarkers for response to PD-1 blockade in *ex vivo* peripheral blood lymphocytes and those that have been completed primarily focus on CD8 T cells [13–18]. Consequently, we sought to identify immune correlates of clinical response following PD-1 blockade by immunophenotyping both CD4 and CD8 T cells in fresh peripheral blood samples. We have previously performed immunophenotyping of circulating lymphocytes in autoimmune settings where we have identified biomarkers of response to immunotherapy [19, 20]. Drawing on this experience, a range of flow cytometry panels were designed and applied to blood samples from patients with advanced malignant melanoma before and after anti-PD-1 therapy.

## Methods and materials

### Patients

Patients with advanced malignant melanoma of cutaneous or mucosal origin were recruited at the Royal Free Hospital NHS Foundation Trust as part of the PASIP research study (NCT02909348). The protocol and consent document of this trial were approved by the London - Bromley Research Ethics Committee (16/LO/1296). All participants were above the age of 18, provided written, informed consent and were deidentified prior to analysis. Patient characteristics of the study cohort are displayed in Supplementary Table S1. Patients were treated with 2 mg/kg of pembrolizumab (humanised monoclonal anti-PD-1, Keytruda, MSD) intravenously on day 1 of each 21-day cycle until progression or unacceptable toxicity developed. Blood samples were typically taken before treatment and then 6 and 12 weeks after treatment initiation. Assessment of patient response was performed prior to cycle 5 infusion by the treating physician according to RECIST version 1.1 [21].

### Sample preparation

Peripheral blood mononuclear cells (PBMCs) were isolated from whole blood by density gradient centrifugation. In brief, whole blood was diluted in phosphate-buffered saline (PBS, Merck) and layered over Histopaque 1077 (Merck) in LeucoSep 50 ml tubes (Greiner Bio-One). After centrifugation at 800 × *g* for 15 minutes with no brake, supernatant above barrier was poured into new 50 ml Falcon tube and centrifuged at 350 × *g* for 10 minutes to remove remaining histopaque. Supernatant was discarded and pellet resuspended in PBS + 2% foetal calf serum (FCS, Life Science Production). Platelets were removed by centrifugation at 250 × *g* for 10 minutes. The resulting pellet was resuspended in PBS + 2% FCS and 1 × 10^6^ cells per flow cytometry panel were used for subsequent flow cytometry staining.

### Flow cytometry

PBMCs were surface stained with four panels of antibody cocktails (see Supplementary Table S2) for 15 minutes at 37°C. For panel 1, cells were then incubated with streptavidin APC for 10 minutes at 4°C. Cells were incubated with fixable viability dye eFluor 780 (Thermo Fisher Scientific) in PBS for 10 minutes at 4°C. For intracellular staining in panel 2, cells were fixed and permeabilized using the Foxp3/Transcription Factor Staining Buffer Set (Thermo Fisher Scientific) and incubated with an antibody cocktail containing anti-human Ki67 Alexa Fluor 488 (clone: Ki-67, Biolegend), CTLA-4 PE (clone: BNI3, BD Biosciences) and Foxp3 APC (clone: 236A/E7, Thermo Fisher Scientific) for 30 minutes at 4°C. Samples were acquired on a BD LSRFortessa (BD Biosciences) using the BD FACSDiva software v.8.0.2 (BD Biosciences).

### Data analysis

For manual analysis, flow cytometry data were analysed using FlowJo v.10 (FlowJo LLC). For unsupervised clustering, pregated live, singlet lymphocytes were preprocessed in R v.4.0.2 as previously described [20] and the FlowSOM algorithm as implemented in the Bioconductor package CATALYST v.1.14.1 was used to identify CD4 and CD8 T cell populations. FlowSOM clustering was then applied again on these T cell populations and optimal number of clusters was identified using delta area plots.

For tSNE projections, flow cytometry data were downsampled to 3000 cells per sample and CATALYST was used to compute tSNE embedding. Principal component analysis (PCA) was performed on scaled and centred data. Prior to PCA, cluster correlation was calculated using Pearson’s *r* and, for cluster pairs that were correlated with an *r* ≥ 0.95, one cluster was randomly removed from analysis. Statistical analysis was performed using R. A two-tailed Student’s *t* test was used to compare two unpaired means. Plots were produced using the CRAN packages ggplot2 v.3.3.5, ggpubr v.0.4.0, ggtext v.0.1.1, cowplot v.1.1.1, scico v.1.3.0, and circlize v.0.4.13 and the Bioconductor packages ComplexHeatmap v.2.6.2. Data cleaning and formatting was carried out using CRAN packages tidyr v.1.1.4, reshape2 v.1.4.4, Rmisc v.1.5, rstatix v.0.7.0, and lubridate v.1.8.0.

### Data sharing statement

Deidentified individual participant data supporting the findings of this study are available from the corresponding author upon request.

## Results

### Incomplete PD-1 blockade on CD4 T cells in non-responders

**Figure 1.**
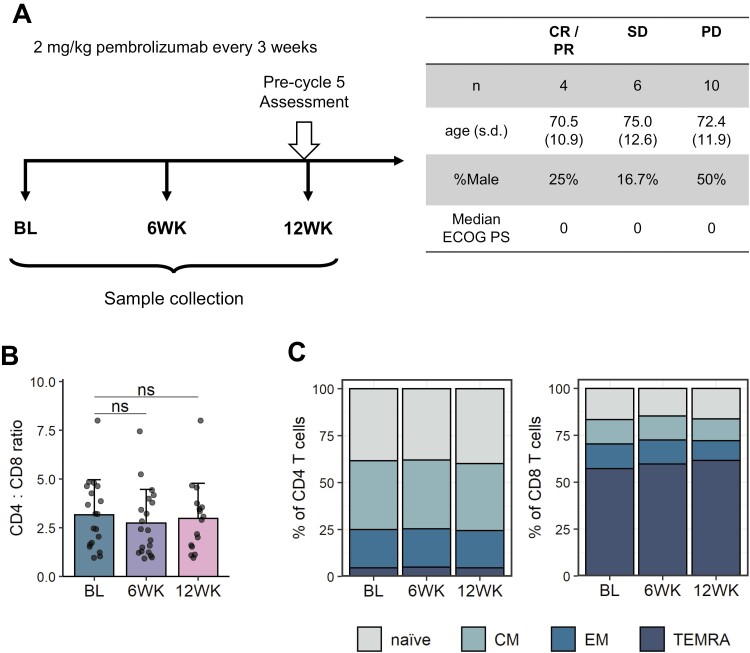
PASIP study cohort. Patients with advanced malignant melanoma were treated with 2 mg/kg pembrolizumab (αPD-1) every 3 weeks. Blood samples were analysed using flow cytometry at baseline and 6 and 12 weeks following treatment initiation. (A) Left: Graphic representation of PASIP study protocol. BL = Baseline, 6WK = 6 weeks, 12WK = 12 weeks. Right: Patient age, sex, and ECOG performance status (PS) across response groups. CR = complete response, PR = partial response, SD = stable disease, PD = progressive disease; s.d., standard deviation; PS, performance status. (B) Ratio of CD4:CD8 T cells at indicated time points. Shown are means + SD. Two-tailed Student’s *t* test; ns, not significant. (C) Distribution of naïve (CD45RA+ CD62L+), central memory (CM, CD45RA- CD62L+), effector memory (EM, CD45RA- CD62L-) and terminally differentiated (TEMRA, CD45RA+ CD62L-) CD4 (left) and CD8 (right) T cells. Shown are means of samples at indicated time points. BL, *n* = 19; 6WK, *n* = 20; 12WK, *n* = 16.

In order to investigate the effect of PD-1 blockade on peripheral immune cells, blood samples from 20 patients with advanced malignant melanoma were collected in the PASIP study. In this study, patients received treatment with pembrolizumab every 3 weeks and blood samples were processed at baseline and 6 and 12 weeks following treatment initiation (**Fig. 1A**). Response was assessed at the pre-cycle 5 (12 weeks) timepoint according to RECIST version 1.1 [21]. In our patient cohort, four patients responded to therapy (one complete responder [CR] and three partial responders [PR]), six patients had an intermediate response (stable disease [SD]) and 10 patients failed to respond to therapy (progressive disease [PD]). The focus of this study was to identify immune correlates of response and we therefore chose to primarily compare patients who responded (CR/PR) and who failed to respond (PD) when stratifying by clinical response. Age, sex, and ECOG performance status were comparable across response groups (**Fig. 1A**).

PBMCs isolated from collected blood samples were analysed using flow cytometry and we first sought to determine whether PD-1 blockade altered the frequencies of CD4 and CD8 T cells, as well as naïve and memory T cell subsets regardless of response. We observed no differences in the CD4:CD8 T cell ratio after treatment with pembrolizumab (**Fig. 1B**). Similarly, frequencies of naïve and memory populations within CD4 and CD8 T cells remained unchanged following PD-1 blockade (**Fig. 1C**, **Supplementary Fig. 1**).

**Figure 2.**
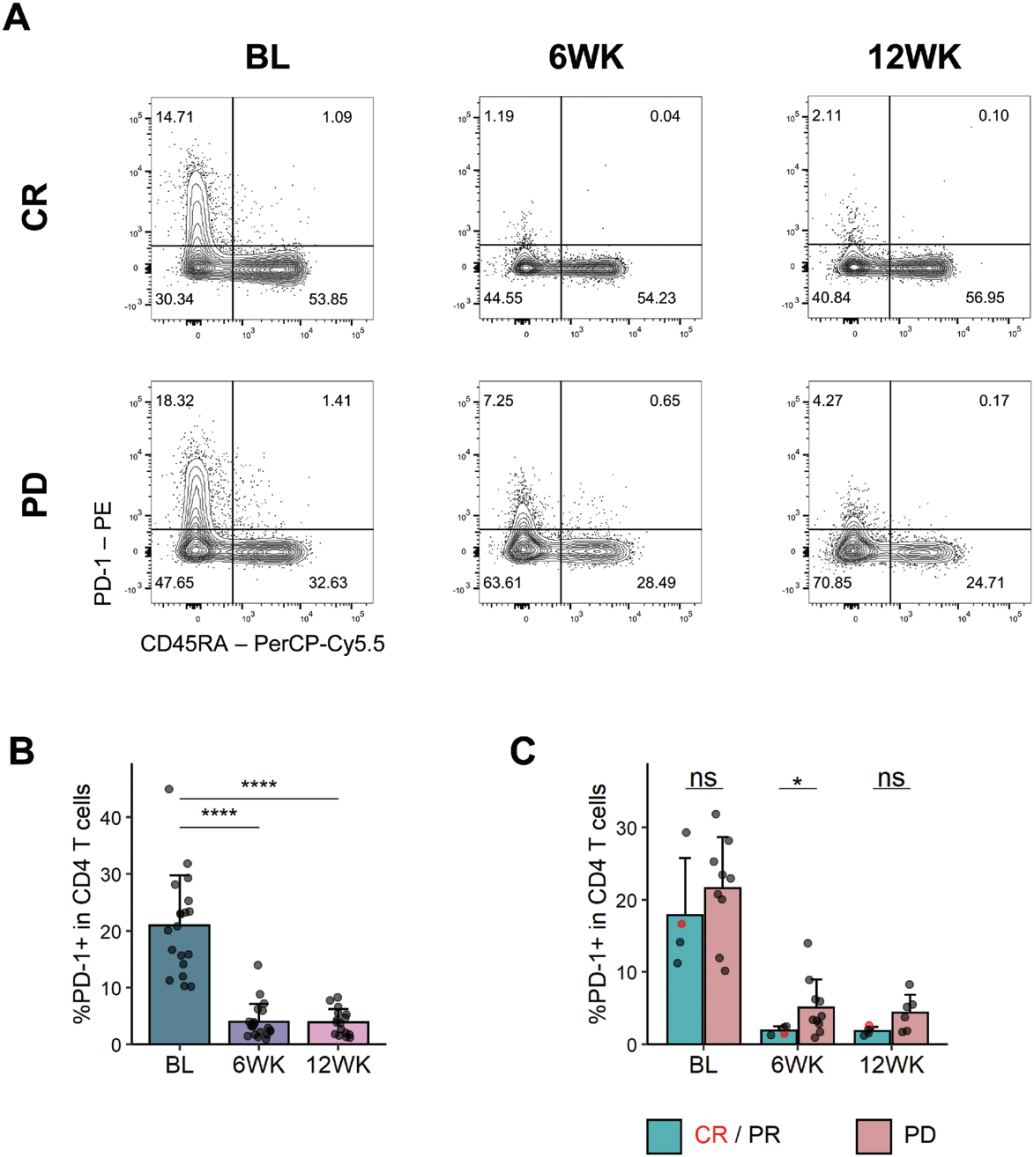
More PD-1 detectable on CD4 T cells in individuals failing to respond to PD-1 blockade. PBMCs were stained with αPD-1 antibodies before and after pembrolizumab treatment. (A) Representative flow cytometry plots for PD-1 and CD45RA staining in CD4 T cells in one CR (top) and one PD (bottom) patient at indicated time points. (B) PD-1+ frequency in CD4 T cells. BL, *n* = 19; 6WK, *n* = 20; 12WK, *n* = 16. (C) PD-1+ frequency in CD4 T cells in responders (CR/PR) and non-responders (PD). CR/PR, *n* = 4 (all time points); PD, *n* = 9 (BL), *n* = 10 (6WK), *n* = 6 (12WK). (B/C) Shown are means + SD. Two-tailed Student’s t test; ****, *P* < 0.0001; *, *P* < 0.05; ns, not significant.

Pembrolizumab precludes binding of the αPD-1 antibody clone J105 used for flow cytometric staining [22], which allowed us to examine occupancy of PD-1 by the therapeutic antibody on peripheral T cells. As expected, PD-1 on CD4 and CD8 T cells was almost undetectable following pembrolizumab treatment (**Fig. 2A and B**, **Supplementary Fig. 2A**). Surprisingly, when we split patients by clinical response, we saw that more PD-1 tended to be detectable on CD4 T cells in patients not responding to therapy, and this reached statistical significance at the 6-week timepoint (**Fig. 2C**, **Supplementary Fig. 2B**). This was not the case for CD8 T cells (**Supplementary Fig. 2C**). The higher residual PD-1 staining in non-responders did not simply reflect higher expression prior to treatment, since patients with the highest expression at 6 weeks were not necessarily those with the highest expression pre-treatment (**Supplementary Fig. 2D**). This suggests the possibility that in non-responders, PD-1 blockade on CD4 T cells may be incomplete for reasons that remain unclear.

### PD-1 blockade induces divergent immune changes in responders and non-responders

**Figure 3.**
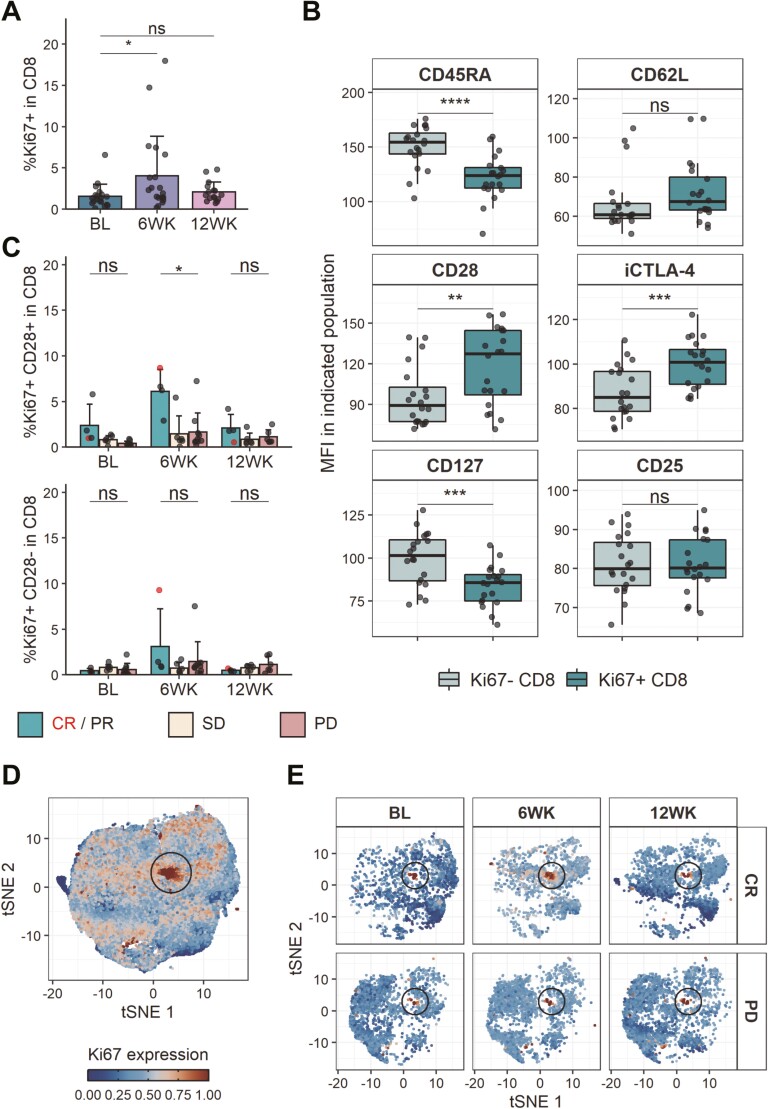
CD28-dependent increase in proliferating CD8 T cells indicative of response following PD-1 blockade. (A) Ki67+ frequency in CD8 T cells. BL, *n* = 19; 6WK, *n* = 20; 12WK, *n* = 16. (B) MFI of indicated markers in Ki67+ or Ki67- CD8 T cells at the 6-week time point. *n* = 20. (C) Frequency of Ki67+ CD28+ (top) and Ki67+ CD28- (bottom) in CD8 T cells stratified by response. CR/PR, *n* = 4 (all time points); SD, *n* = 6 (all time points); PD, *n* = 9 (BL), *n* = 10 (6WK), *n* = 6 (12WK). (D) tSNE projection of downsampled and pooled CD8 T cells of CR, PR, and PD samples. Colour indicates scaled Ki67 expression. (E) tSNE projection shown in (D) of one CR and one PD sample at indicated time points. Colour indicates scaled Ki67 expression. (A/C) Shown are means + SD. (B) Shown are box plots, with black horizontal line denoting median value, while box represents the IQRs (IQR, Q1–Q3 percentile) and whiskers show the minimum (Q1 − 1.5× IQR) and maximum (Q3 + 1.5× IQR) values. (A/B/C) Two-tailed Student’s *t* test; ****, *P* < 0.0001; ***, *P* < 0.001; **, *P* < 0.01; *, *P* < 0.05; ns, not significant.

PD-1 engagement suppresses T cell responses by dampening signals necessary for efficient T cell stimulation [23]. Accordingly, blockade of PD-1 should enhance T cell activation and proliferation. We therefore evaluated expression of the proliferation marker Ki67 in T cells following pembrolizumab treatment. As expected, Ki67 expression was significantly increased in CD8 T cells 6 weeks following pembrolizumab treatment initiation (**Fig. 3A**). There was also a trend towards increased proliferation in CD4 T cells, however, this was not statistically significant (**Supplementary Fig. 3A**). To further characterise the proliferating CD8 T cells, expression levels of several markers were compared between the Ki67+ and Ki67- fractions. Ki67+ CD8 T cells expressed higher intracellular CTLA-4 and lower CD45RA and CD127 levels than the non-proliferating CD8 T cells (**Fig. 3B**). Moreover, CD28 expression was higher on Ki67+ CD8 T cells when compared with Ki67- CD8 T cells. While PD-1 can inhibit T cell responses by suppressing TCR signalling [24, 25], there is also evidence that PD-1 engagement can lead to the dephosphorylation of CD28, thereby dampening CD28 downstream signalling [26–28]. Stratification of CD28+Ki67+ and CD28-Ki67+ CD8 T cells by clinical response revealed that there was a significantly higher frequency of CD28+Ki67+ CD8 T cells in responders than in non-responders at the 6-week timepoint, with the CR patient showing the highest levels of Ki67 (**Fig. 3C–E**, **Supplementary Fig. 3B**). While a trend for increased Ki67 expression in responders at the 6-week timepoint was also observable when looking at the whole CD8 T cell population this did not reach statistical significance (**Supplementary Fig. 3C**). Ki67 expression in CD4 T cells also did not differ between clinical response groups (**Supplementary Fig. 3D**). Taken together, these data suggest that response following PD-1 blockade is marked by increased proliferation in CD28-expressing CD8 T cells.

**Figure 4.**
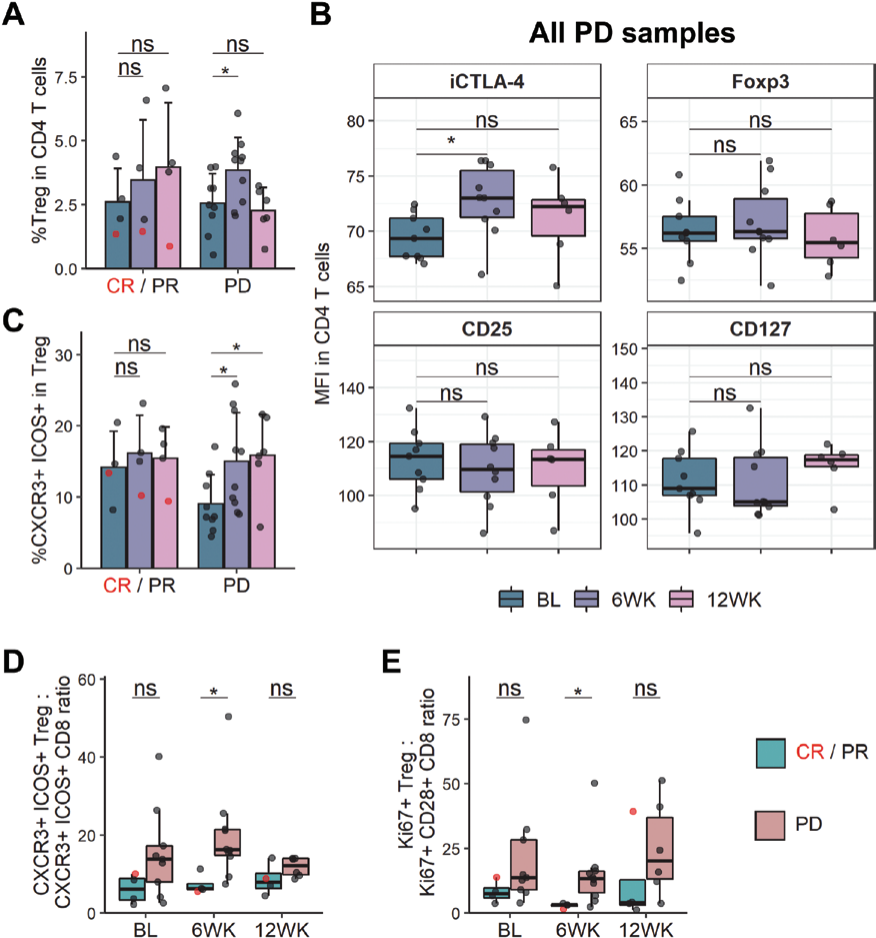
Ratio of activated and proliferating Treg and CD8 T cells is skewed in non-responders. (A) Frequency of Treg (CD25+ CD127- Foxp3+ CTLA-4+) in CD4 T cells in responders and non-responders. (B) MFI of CD25, CD127, Foxp3, and intracellular CTLA-4 in CD4 T cells of non-responders (PD). (C) CXCR3+ ICOS+ frequency in Treg (CD25+ CD127-). (D) Ratio of CXCR3+ ICOS+ frequency in Treg to CXCR3+ ICOS+ frequency in CD8 T cells. (E) Ratio of Ki67+ frequency in Treg to Ki67+ CD28+ frequency in CD8 T cells. (A/C) Shown are means + SD. (B/D/E) Shown are box plots, with black horizontal line denoting median value, while box represents the IQRs (IQR, Q1–Q3 percentile) and whiskers show the minimum (Q1 − 1.5× IQR) and maximum (Q3 + 1.5× IQR) values. CR/PR, *n* = 4 (all time points); PD, *n* = 9 (BL), *n* = 10 (6WK), *n* = 6 (12WK). Two-tailed Student’s *t* test; *, *P* < 0.05; ns, not significant.

We were next interested in identifying changes occurring specifically in non-responders to pembrolizumab treatment. When investigating CD4 T cell subsets, we found that in patients that did not respond to therapy, the frequency of regulatory T cells (Treg, CD25+CD127-Foxp3+CTLA-4+) transiently increased 6 weeks post-treatment initiation and then returned to baseline by the 12-week timepoint (**Fig. 4A**, **Supplementary Fig. 4A**). Interestingly, when evaluating expression of Treg markers in non-responder samples using median fluorescence intensity (MFI) in all CD4 T cells, we saw that only expression of CTLA-4 increased significantly following pembrolizumab treatment, while other markers, including Foxp3, CD25, and CD127 remained consistent over the treatment period (**Fig. 4B**). This increase was also observed when analysing gated Treg (**Supplementary Fig. 4B and C**). Additionally, Treg in non-responders but not responders showed increased expression of CXCR3 and ICOS after therapy (**Fig. 4C**, **Supplementary Fig. 4D**) and there was also a trend towards increased Ki67 expression in the Treg compartment (**Supplementary Fig. 4E**). Therefore, an increase in CTLA-4 expressing Treg is associated with non-response following PD-1 blockade.

As frequencies of immune cell populations can have limited applicability when clinically evaluating response to immunotherapy in individual patients, we sought to evaluate whether assessing ratios of certain immune cell subsets would provide a more robust approach for stratifying patient responses. Since we saw increases in activated and proliferating Treg (CXCR3+ICOS+ and Ki67+, respectively) in non-responders following PD-1 blockade and conversely higher frequencies of corresponding CD8 T cells in responders (**Fig. 3**; **Supplementary Fig. 5A**), we investigated whether Treg:CD8 ratios would provide a way to distinguish clinical response. Both the CXCR3+ICOS+ Treg to CXCR3+ICOS+ CD8 T cell ratio and Ki67+ Treg to Ki67+CD28+ CD8 T cell ratio were skewed to being significantly higher in non-responders 6 weeks following pembrolizumab treatment initiation (**Fig. 4D and E**, **Supplementary Fig. 5B and C**). Specifically, an average ratio of 1 CXCR3+ICOS+ CD8 T cell to 19.8 CXCR3+ICOS+ Treg was observed in non-responders while responders showed an average ratio of 1 to 7.3. The ratio of Ki67+CD28+ CD8 T cells to Ki67+ Treg was 1 to 14.9 in non-responders and 1 to 2.7 in responders. Importantly, this does not simply reflect the overall Treg:CD8 ratio which was not significantly different between responders and non-responders at all timepoints investigated (**Supplementary Fig. 5D**). Therefore, assessing the ratios of activated and proliferating Treg and CD8 T cells may provide a way to stratify clinical response following pembrolizumab treatment. Additionally, we found that CXCR3+ICOS+ Treg and CD8 T cells positively correlated with their Ki67 expressing counterparts at the 6-week timepoint (**Supplementary Fig. 5E and F**), suggesting that CXCR3 and ICOS co-expression could potentially be used as a surrogate marker for proliferation in these cell types.

### Clinical response following PD-1 blockade can be distinguished at baseline

Results presented thus far focus on response-specific changes occurring in peripheral T cells following PD-1 blockade, particularly 6 weeks post-treatment initiation. However, in many malignancies, the therapeutic window is limited and biomarkers to predict patient response to immunotherapy before treatment would be highly beneficial. Accordingly, we were interested to see whether we could identify immune signatures in baseline bleeds that could stratify patients based on their clinical response following PD-1 blockade.

In order to maximise the information obtained from the flow cytometry data, we decided to use the unsupervised clustering algorithm FlowSOM [29] to identify as many immune cell subsets as possible. Across the four flow cytometry panels used for analysis, a total of 156 CD4 and CD8 T cell clusters were detected (**Supplementary Fig. 6**). Following pairwise correlation comparison, four of these clusters were found to be highly correlated (Pearson correlation coefficient > 0.95) to other clusters and therefore removed. This left 152 clusters which were used to conduct principal component analysis (PCA). We reasoned that patient age may also influence clinical response and thus included this as a feature in the analysis as well.

**Figure 5.**
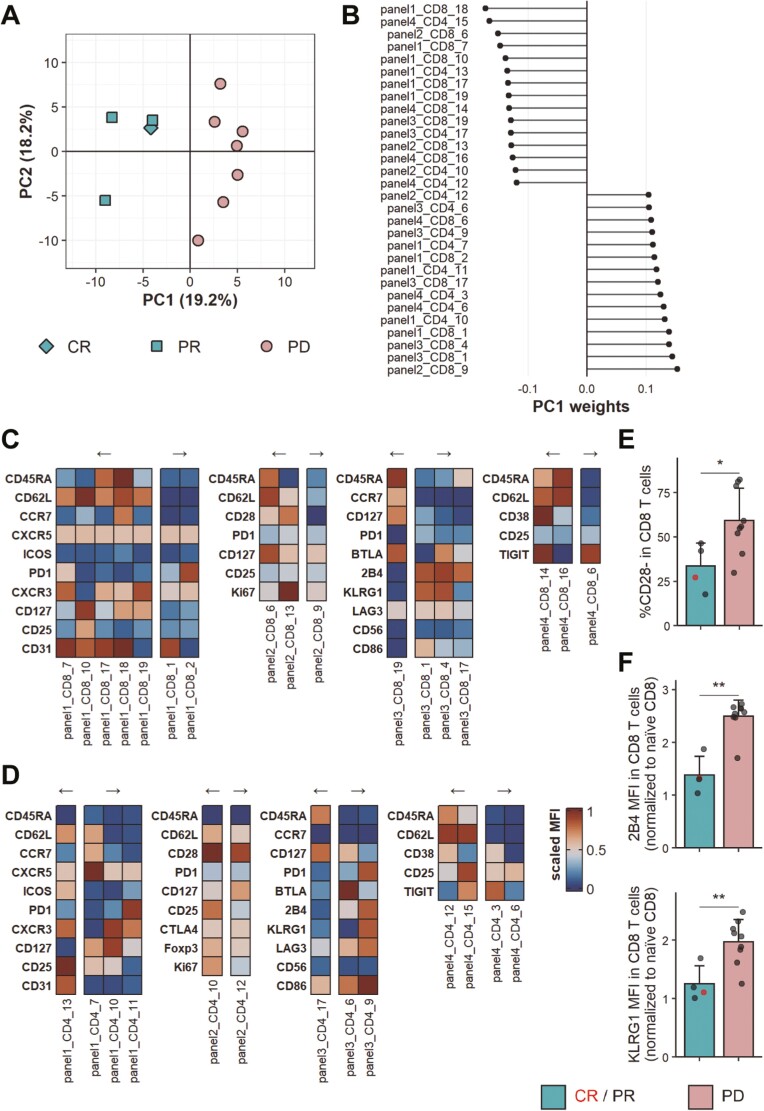
Clinical response following PD-1 blockade can be distinguished at baseline. FlowSOM clustering was applied to CD4 and CD8 T cells stained with four distinct flow cytometry panels. (A) PCA on FlowSOM clusters of baseline samples of responders and non-responders. CR, *n* = 1; PR, *n* = 3; PD, *n* = 7. (B) Top and bottom 15 FlowSOM clusters contributing to PC1 ordered by PC weight. (C) Heatmaps showing scaled MFI of indicated markers in CD8 T cell clusters shown in (B). (D) Heatmaps showing scaled MFIs of indicated markers in CD4 T cell clusters shown in (B). Arrows in (C) and (D) indicate directionality of associated PC1 weight. (E) CD28- frequency in CD8 T cells in baseline samples of responders and non-responders. (F) MFI of 2B4 (top) and KLRG1 (bottom) in CD8 T cells normalised to MFI in naïve (CD45RA+ CCR7+) CD8 T cells. Data shown is from baseline samples of responders and non-responders. (E/F) Shown are means + SD. CR/PR, *n* = 4; PD, *n* = 9. Two-tailed Student’s *t* test; **, *P* < 0.01; *, *P* < 0.05.

Remarkably, baseline bleeds of responders and non-responders showed a clear separation along the first principal component (PC1), which accounts for 19.2% of the variance in this dataset (**Fig. 5A**). We investigated the top 15 clusters contributing to PC1 in either direction to identify characteristics of the T cell populations driving this separation (**Fig. 5B–D**). One of the populations contributing most to PC1 comprised CD8 T cells lacking expression of CD28 (panel2_CD8_9). Indeed, after manually gating CD28 expression, we saw that on average more than 50% of CD8 T cells in patients that failed to respond were CD28- at baseline, while this was only the case for about 25% of CD8 T cells in the patients who responded to therapy (**Fig. 5E**, **Supplementary Figs. 3B and 7A**). Conversely, one of the clusters associated with response showed high Ki67 expression in CD28+ CD8 T cells (panel2_CD8_13). It is important to note that this cluster was highly correlated with a Ki67 expressing CD4 conventional T cell cluster (panel2_CD4_16, **Supplementary Fig. 7B and C**) which was consequently removed from the features used for PCA. This, therefore, indicates that patients responding to PD-1 blockade show higher baseline proliferation in CD8 as well as CD4 T cells.

Furthermore, we noted that clusters associated with non-response to therapy predominantly exhibited an effector memory or terminally differentiated effector memory phenotype, while clusters linked to response tended to be central memory or naïve T cells. Among the latter, a CD45RA+CD62L+ CD8 T cell cluster (panel4_CD8_14) caught our attention, since it exhibited high CD38 expression and was associated with response following therapy. We could confirm by manual gating that CD38hiCD62L+ CD8 T cells were indeed enriched in responders at baseline (**Supplementary Fig. 7D**). Of possible interest, this population also tended to be higher in frequency in responders compared with non-responders at the 6- and 12-week timepoints and displayed a remarkable increase in the patient showing a complete response at the 6-week timepoint (**Supplementary Fig. 7D and E**). Finally, two CD8 T cell clusters associated with non-response following pembrolizumab treatment showed high expression of the inhibitory receptors 2B4 and KLRG1 (panel3_CD8_1 and panel3_CD8_4). Indeed, the MFI of these two receptors in CD8 T cells was significantly higher in non-responders when compared with responders at baseline (**Supplementary Fig. 8A and B**, **Supplementary Fig. 9**). Since the MFI is subjective to the fluorochrome and flow cytometer used, we opted to normalise using the MFI of 2B4 and KLRG1 on naïve CD8 T cells, where it was equivalent between responders and non-responders (**Supplementary Fig. 8C**). Using this approach, we saw a 2.5× increase in 2B4 expression and 2× increase in KLRG1 expression in all CD8 T cells compared with naïve CD8 T cells of non-responders, while there was only a 1.4× and 1.3× increase, respectively, in the responders (**Fig. 5F**, **Supplementary Fig. 8D**).

Taken together, these results suggest that it may be possible to gain insights into the clinical response following pembrolizumab treatment by utilising flow cytometric analysis of CD4 and CD8 T cell subsets at baseline. Specifically, higher baseline proliferation in CD4 and CD8 T cells is associated with a response to therapy, while non-response is associated with a CD8 T cell compartment featuring more CD28-negative and inhibitory receptor-expressing cells.

## Discussion

While checkpoint inhibitor therapies are now an established treatment option in cancer therapy, clinical response of patients is variable. Robust biomarkers of response following PD-1 blockade are still lacking and peripheral blood immunophenotyping remains incompletely explored. Using T cell-focussed flow cytometric analysis we have identified a number of features that correlate with response to pembrolizumab in patients with advanced malignant melanoma.

We found that patients that responded to PD-1 blockade experienced a greater increase in expression of the proliferation marker Ki67 particularly in CD28-expressing CD8 T cells. This is in accordance with previously published data showing an increase in CD8 T cell proliferation that correlates with clinical response and is largely driven by CD28-expressing CD8 T cells [13, 27]. Furthermore, we show that the frequency of proliferating CD28+ CD8 T cells directly correlates with the frequency of CXCR3- and ICOS-expressing CD8 T cells at the 6-week timepoint, suggesting that the latter could be used as surrogate markers for PD-1 induced proliferation, avoiding the need for intracellular Ki67 staining. ICOS expression on CD8 T cells responding to PD-1 blockade has previously been shown [13], while work by Chow *et al*. demonstrated that CXCR3 expression is important for αPD-1 efficacy in a mouse tumour model [30].

Conversely, patients that failed to respond to PD-1 blockade showed a transient increase in Treg 6 weeks after treatment initiation. It has previously been shown that more Treg and a higher frequency of proliferating Treg can be found in peripheral blood of melanoma patients when compared with healthy controls [12, 31, 32] and presence of Treg within the tumour is associated with a worse prognosis [33], consistent with the idea that Treg suppress anti-tumour immune responses [34]. The response of CD8 T cells to PD-1 blockade has been extensively studied, however evidence of modulation of Treg has only recently started to emerge. While initial data did not show any correlation between increases in Treg and clinical outcome following PD-1 blockade [12], later reports showed that presence of proliferating Treg within the tumour as well as peripheral blood following PD-1 blockade was associated with a poor prognosis [35, 36], consistent with our findings. The difference in observations may be attributed to the markers used to identify Treg in these studies, with our data showing that it is particularly Treg expressing CTLA-4 that are increased following therapy in non-responders. This upregulation of CTLA-4 on Treg could be a result of enhanced activation following PD-1 blockade and higher levels of CTLA-4 are associated with increased Treg function [37]. CTLA-4 expression is also a key feature of highly suppressive Treg found to be enriched in tumour tissue of breast and lung cancer patients, which are associated with more aggressive disease [38–40]. Of note, recent analysis has highlighted the potential for CTLA-4-expressing tumour-infiltrating follicular regulatory T cells (Tfr) to limit the effectiveness of PD-1 inhibitors [41]. Together, these studies provide strong biological context to our finding that non-responders are characterised by increases in CTLA-4+ Treg. Since CTLA-4 is a key mediator of Treg suppression [42], it is possible that inhibition of Treg function by anti-CTLA-4 antibodies might prevent expanded Treg from limiting anti-tumour immunity in these individuals, perhaps explaining why therapies targeting both PD-1 and CTLA-4 work well together.

In our pre-treatment analysis, we identified that responders showed a higher frequency of proliferating CD28-expressing CD8 T cells as well as proliferating CD4 conventional T cells, suggestive of ongoing immunity, potentially encompassing anti-tumour immune responses. Conversely, non-responders had higher frequencies of CD28- CD8 T cells, that are less responsive to PD-1 therapy, and showed higher expression of inhibitory receptors 2B4 and KLRG1 at baseline. CD28 expression on CD8 T cells is known to be downregulated by repeated antigen stimulation and CD28- CD8 T cells accumulate with age [43, 44]. Both 2B4 and KLRG1 have previously been linked to CD8 T cell exhaustion in chronic viral infection [45, 46] and targeting KLRG1 in combination with PD-1 blockade has been evaluated in an *in vivo* cancer model [47]. Interestingly, recent work has identified a population of CD8 T cells with immunoregulatory function that have lost CD28 and express both 2B4 and KLRG1 [48]. It is possible that the non-responders in our cohort had higher levels of these suppressive CD8 T cells prior to therapy.

While complete responses following PD-1 pathway blockade have been observed, they only occur in a limited number of individuals. Biomarkers to identify this patient group would therefore be highly beneficial. In our patient cohort, only one individual showed a complete response to pembrolizumab, precluding us from further investigating correlates of response. However, we did observe a notable increase in CD38hiCD62L+ CD8 T cells 6 weeks after treatment initiation in this patient. This cell subset bears some resemblance to the circulating exhausted CD8 T cells shown by Huang *et al*. to be reinvigorated following PD-1 blockade [12] and could conceivably demark patients responding exceptionally well to therapy.

Finally, our anti-PD-1 antibody staining revealed a higher frequency of unoccupied PD-1 on CD4 T cells at the 6-week timepoint in non-responders. Most reports evaluate PD-1 expression using antibodies targeting the Fc region of the relevant therapeutic antibody and there is relatively little data evaluating PD-1 occupancy in patients following treatment. Data from Das *et al*. showed that even when PD-1 is entirely occupied by therapeutic antibody in peripheral blood, blockade of PD-1 on TILs is still incomplete [49]. This analysis could suggest that if PD-1 blockade is not complete in the periphery, blockade within the tumour may not be sufficient either, potentially affecting treatment efficacy. Additionally, Zappasodi *et al*. made use of an αPD-1 antibody that is not blocked by therapeutic αPD-1 antibodies to show that PD-1hi CD4 T cells are decreased following PD-1 blockade and that this reduction was less prevalent in patients that failed to respond to therapy [50]. While we cannot directly compare these PD-1hi CD4 T cells with the PD-1+ CD4 T cells we observe after therapy in non-responders, our data suggest that the relationship between treatment efficacy and PD-1 receptor occupancy warrants further investigation.

In summary, by conducting peripheral blood immunophenotyping in patients with advanced malignant melanoma treated with pembrolizumab, we identified skewing of the peripheral immune response both at baseline and shortly after treatment initiation that correlated with response following PD-1 blockade. Specifically, patients that failed to respond to therapy displayed a more immune suppressive phenotype with low CD28 expression on CD8 T cells and an increase in Treg following therapy. Additionally, we found that ratios of activated and proliferating Treg and CD8 T cells could stratify patients by clinical response. Our results were obtained in a limited number of patients, and it will be important to validate these findings in larger patient cohorts. Nonetheless, our work suggests that T cell-directed flow cytometric assays could provide a further tool to inform rapid treatment decisions.

## Supplementary material

Supplementary data are available at *Immunotherapy Advances* online.

Figure S1. Frequencies of naïve and memory CD4 and CD8 T cells does not change following PD-1 blockade. (A) Frequency of naïve (top left, CD45RA+ CD62L+), central memory (CM, top right, CD45RA- CD62L+), effector memory (EM, bottom left, CD45RA- CD62L-) and terminally differentiated (TEMRA, bottom right, CD45RA+ CD62L-) in CD4 T cells. (B) Frequency of naïve (top left, CD45RA+ CD62L+), central memory (CM, top right, CD45RA- CD62L+), effector memory (EM, bottom left, CD45RA- CD62L-) and terminally differentiated (TEMRA, bottom right, CD45RA+ CD62L-) in CD8 T cells. Shown are means + s.d.. BL, n = 19; 6WK, n = 20; 12WK, n = 16. Two-tailed Student’s t test; ns, not significant.

Figure S2. PD-1 detection in CD8 T cells following PD-1 blockade is the same in responders and non-responders. (A) PD-1+ frequency in CD8 T cells. BL, n = 19; 6WK, n = 20; 12WK, n = 16. (B) PD-1+ frequency in CD4 T cells stratified by response. (C) PD-1+ frequency in CD8 T cells stratified by response. (D) (top) PD-1+ frequency in CD4 T cells in CR, PR, SD and PD patients. Points from same patient are connected by lines. Colour indicates PD-1+ frequency at baseline. (bottom) Ranking of PD-1 expression in CR, PR, SD and PD patients at baseline and the 6-week timepoint. For P23 no baseline bleed was available. (B/C/D) CR/PR, n = 4 (all time points); SD, n = 6 (all time points); PD, n = 9 (BL), n = 10 (6WK), n = 6 (12WK). Shown are means + s.d.. Two-tailed Student’s t test; ****, P < 0.0001; *, P < 0.05; ns, not significant.

Figure S3. Increase in proliferation in CD4 T cells does not distinguish responders and non-responders. (A) Ki67+ frequency in CD4 T cells. (B) Representative flow cytometry plots showing Ki67 and CD28 expression in CD8 T cells in baseline, 6-week and 12-week bleeds of one PR and one PD patient. (C) Ki67+ frequency in CD8 T cells stratified by ­response. (D) Frequency of Ki67+ (left), Ki67+ CD28+ (middle) and Ki67+ CD28- (right) in CD4 T cells stratified by response. (A) BL, n = 19; 6WK, n = 20; 12WK, n = 16. (C/D) CR/PR, n = 4 (all time points); SD, n = 6 (all time points); PD, n = 9 (BL), n = 10 (6WK), n = 6 (12WK). Shown are means + s.d.. Two-tailed Student’s t test; ns, not significant.

Figure S4. CTLA-4+ Treg are transiently increased in non-responders following PD-1 blockade. (A) Frequency of Treg (CD25+ CD127- Foxp3+ CTLA-4+) in CD4 T cells. (B) MFI of intracellular CTLA-4 in Treg (CD25+ CD127-) of non-responders. (C) Scaled histogram showing intracellular CTLA-4 expression in naïve T cells (filled) or Treg (open) from a non-responder at the indicated time points. (D) CXCR3+ ICOS+ frequency in Treg (CD25+ CD127-). (E) Ki67+ frequency in Treg. (A/D/E) Shown are means + s.d.. (B) Shown are box plots, with black horizontal line denoting median value, while box represents the IQRs (IQR, Q1–Q3 percentile) and whiskers show the minimum (Q1 − 1.5× IQR) and maximum (Q3 + 1.5× IQR) values. (A/B/D/E) CR/PR, n = 4 (all time points); SD, n = 6 (all time points); PD, n = 9 (BL), n = 10 (6WK), n = 6 (12WK). Two-tailed Student’s t test; *, P < 0.05; ns, not significant.

Figure S5. CXCR3 and ICOS expression correlates with proliferation of Treg and CD8 T cells. (A) CXCR3+ ICOS+ frequency in CD8 T cells. (B) Ratio of CXCR3+ ICOS+ frequency in Treg to CXCR3+ ICOS+ frequency in CD8 T cells. (C) Ratio of Ki67+ frequency in Treg to Ki67+ CD28+ frequency in CD8 T cells. (D) Ratio of Treg frequency (CD25+ CD127- Foxp3+ CTLA-4+) to CD8 T cell frequency. (E) Pearson correlation of Ki67+ frequency in Treg (CD25+ CD127- Foxp3+ CTLA-4+) to CXCR3+ ICOS+ frequency in Treg (CD25+ CD127-). (F) Pearson correlation of Ki67+ frequency to CXCR3+ ICOS+ frequency in CD8 T cells. (A) Shown are means + s.d.. (B/C/D) Shown are box plots, with black horizontal line denoting median value, while box represents the IQRs (IQR, Q1–Q3 percentile) and whiskers show the minimum (Q1 − 1.5× IQR) and maximum (Q3 + 1.5× IQR) values. (A/B/C/D) CR/PR, n = 4 (all time points); SD, n = 6 (all time points); PD, n = 9 (BL), n = 10 (6WK), n = 6 (12WK). Two-tailed Student’s t test; ***, P < 0.001; *, P < 0.05; ns, not significant. (E/F) CR, n = 1; PR, n = 3; SD, n = 6; PD, n = 10. Pearson’s R and associated p value are depicted on plots. Black line only for visualisation purposes.

Figure S6. Heatmaps of maker expression in FlowSOM clusters. FlowSOM clustering was applied to CD4 and CD8 T cells stained with four distinct flow cytometry panels. Shown are heatmaps of scaled MFIs of indicated markers in CD4 (left) and CD8 (right) T cells.

Figure S7. Clinical response following PD-1 blockade can be distinguished using baseline bleeds. (A) CD28- frequency in CD8 T cells in baseline samples. (B) Pearson correlation of frequencies of FlowSOM clusters panel2_CD4_16 and panel2_CD8_13. (C) Pearson correlation of manually gated Ki67+ frequency in Tconv (non-Treg) and Ki67+ CD28+ frequency in CD8 T cells. (D) CD62L+ CD38hi frequency in CD8 T cells. (E) Flow cytometry plots showing CD62L and CD38 expression in CD8 T cells in baseline and 6-week bleeds of CR patient. (A/D) Shown are means + s.d.. CR/PR, n = 4 (all time points); SD, n = 6 (all time points); PD, n = 9 (BL), n = 10 (6WK), n = 6 (12WK). Two-tailed Student’s t test; **, P < 0.01; *, P < 0.05; ns, not significant. (B/C) CR, n = 1; PR, n = 3; SD, n = 6; PD, n = 7. Pearson’s R and associated p value are depicted on plots. Black line only for visualisation purposes.

Figure S8. CD8 T cells of non-responders have higher expression of 2B4 and KLRG1 at baseline. (A) MFI of 2B4 (left) and KLRG1 (right) in CD8 T cells in baseline samples. (B) Representative flow cytometry plots showing 2B4 and KLRG1 expression in CD8 T cells in baseline bleed of one PR patient and one PD patient. (C) MFI of 2B4 (left) and KLRG1 (right) in naïve (CD45RA+ CCR7+) CD8 T cells in baseline samples. (D) MFI of 2B4 (left) and KLRG1 (right) in CD8 T cells normalised to MFI in naïve (CD45RA+ CCR7+) CD8 T cells. Data shown is from baseline samples. (A/C/D) Shown are means + s.d.. CR/PR, n = 4; SD, n = 6; PD, n = 9. Two-tailed Student’s t test; **, P < 0.01; ns, not significant.

Figure S9. Gating strategies. Shown are representative gating strategies for relevant CD4 and CD8 T cell populations in indicated flow cytometry panels. For all panels, cells in first gate are live, singlet Lymphocytes. Grey background indicates same parent gate.

Table S1. Patient characteristics. Shown are characteristics of patients enrolled in the PASIP study.

Table S2. Flow cytometry surface panels. Shown are the four flow cytometry panels of antibodies that were used for surface staining of PBMCs in this study.

ltad001_suppl_Supplementary_Figures

ltad001_suppl_Supplementary_Table_S1

ltad001_suppl_Supplementary_Table_S2

## Data Availability

Deidentified individual participant data supporting the findings of this study are available from the corresponding author upon request.

## Abbreviations

CR: Complete responder
FCS: Foetal calf serum
MFI: Median fluorescence intensity
PBMC: Peripheral blood mononuclear cell
PBS: Phosphate-buffered saline
PCA: Principal component analysis
PD: Progressive disease
PR: Partial responder
SD: Stable disease
Tfr: Follicular regulatory T cells
TIL: Tumour-infiltrating lymphocyte
Treg: Regulatory T cells

## Permission to reproduce

Not applicable to this manuscript.

## Clinical trial registration

The PASIP research study is listed at ClinicalTrials.gov and can be found under the identifier NCT02909348. The protocol and consent document of this trial were approved by the London - Bromley Research Ethics Committee (16/LO/1296).

## ARRIVE guidelines

Not applicable to this manuscript.
