# Supplementary material for: Stratification of PD-1 blockade response in melanoma using pre- and post-treatment immunophenotyping of peripheral blood
Source: Immunother Adv. Author manuscript; Available in PMC 2023 Feb 17. (PMC9929715; doi:10.1093/immadv/ltad001)
Supplement: Supplementary Material [file EMS163170-supplement-Supplementary_Material.pdf]

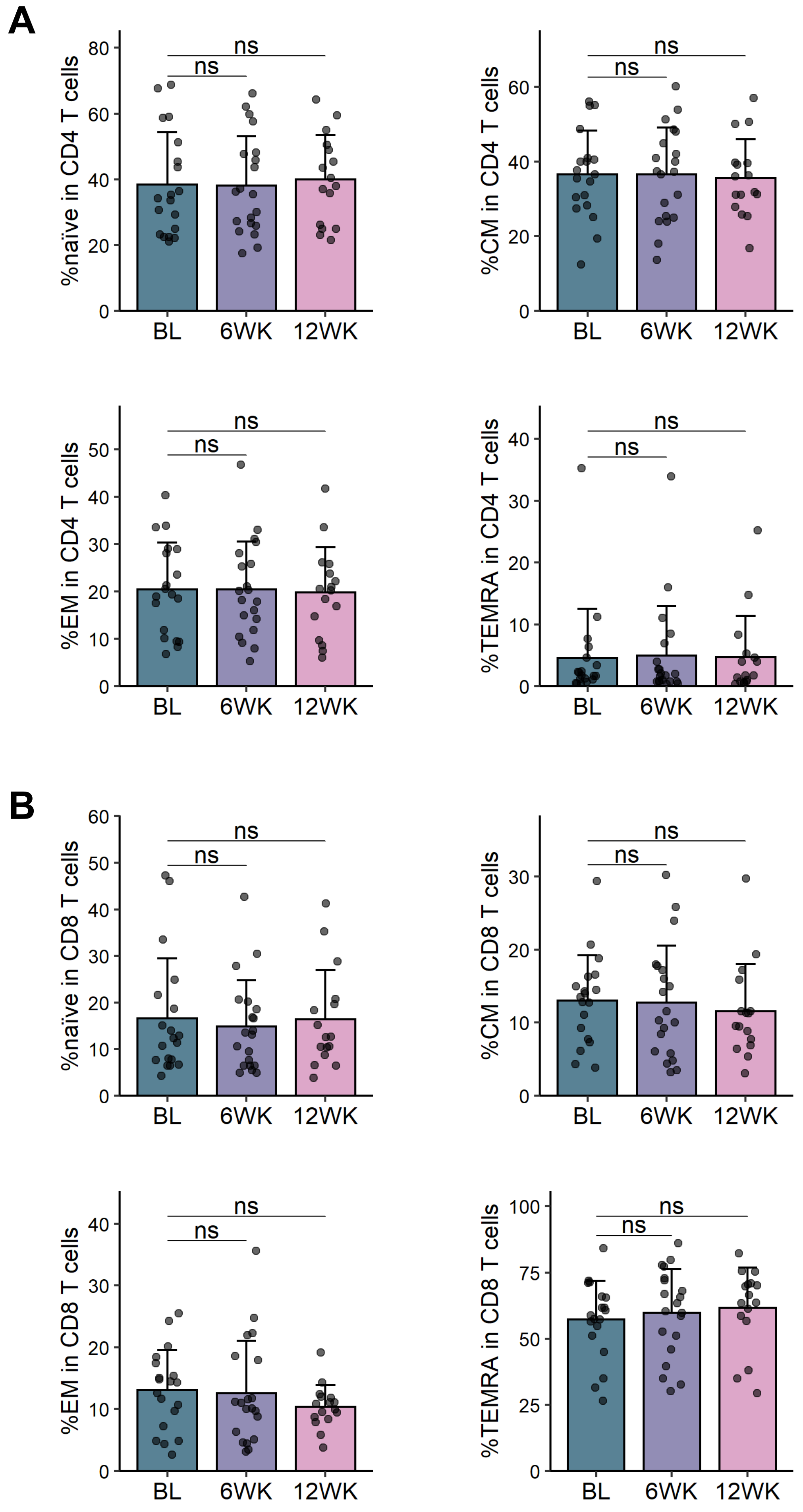

Suppl. Figure 1

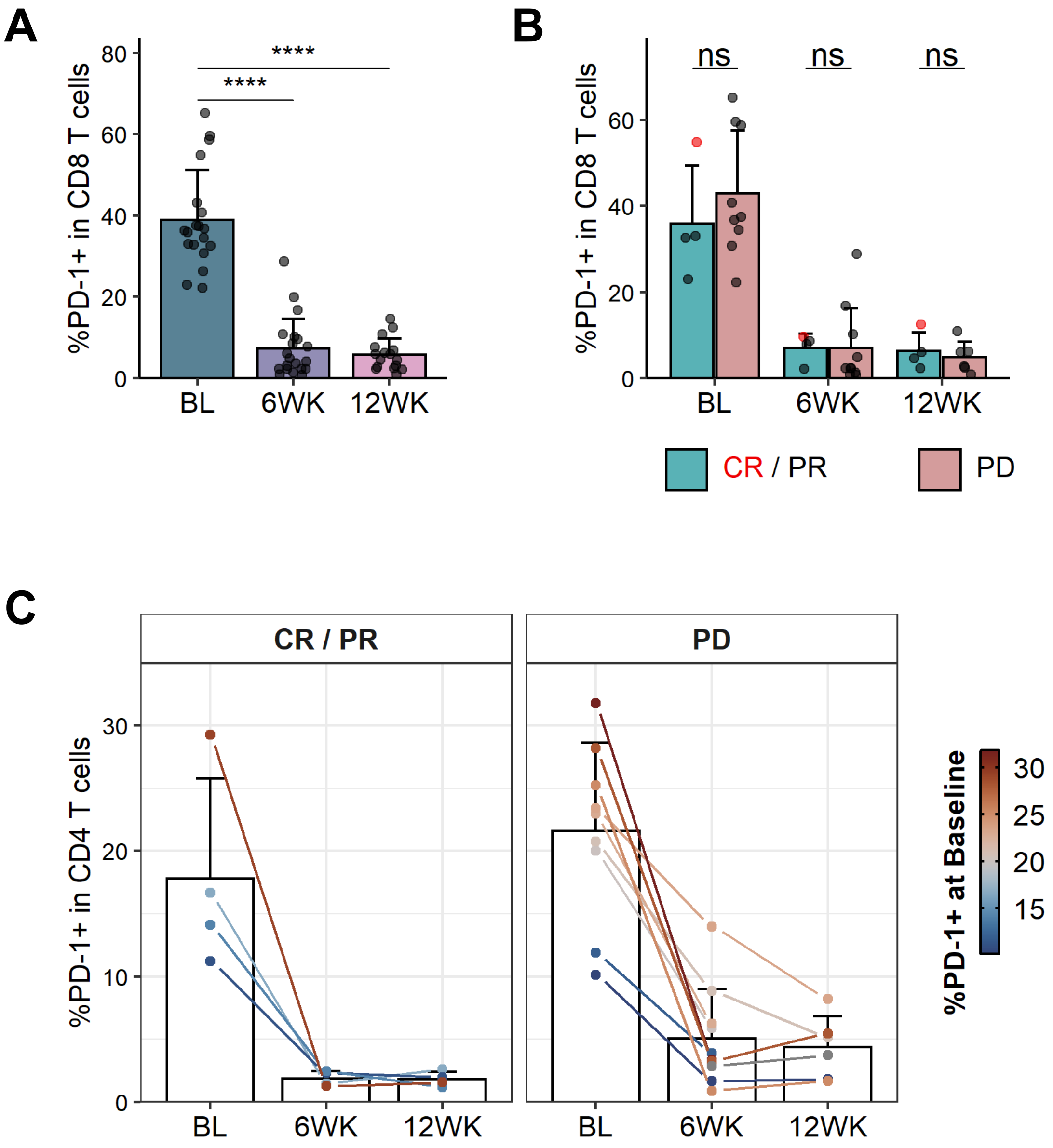

Patient	P19	P25	P22	P24	P16	P15	P11	P07	P05	P20	P02	P12	P03	P23
Response	PD	PR	PD	PD	PD	PD	PD	PD	CR	PR	PD	PR	PD	PD
Rank BL	1	2	3	4	5	6	7	8	9	10	11	12	13	-
Rank 6WK	6	13	7	14	1	3	2	4	12	9	5	10	11	8

Suppl. Figure 2

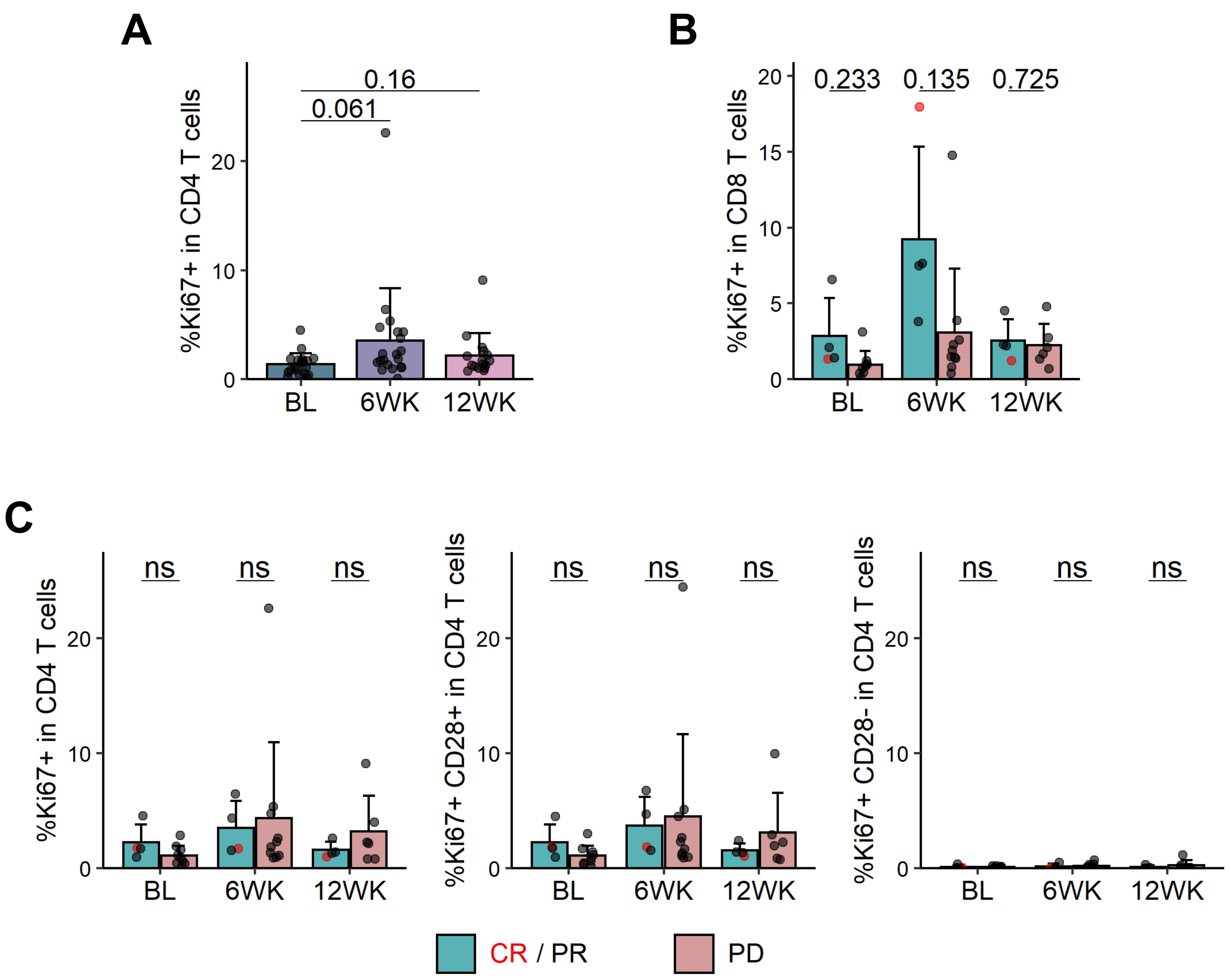

Suppl. Figure 3

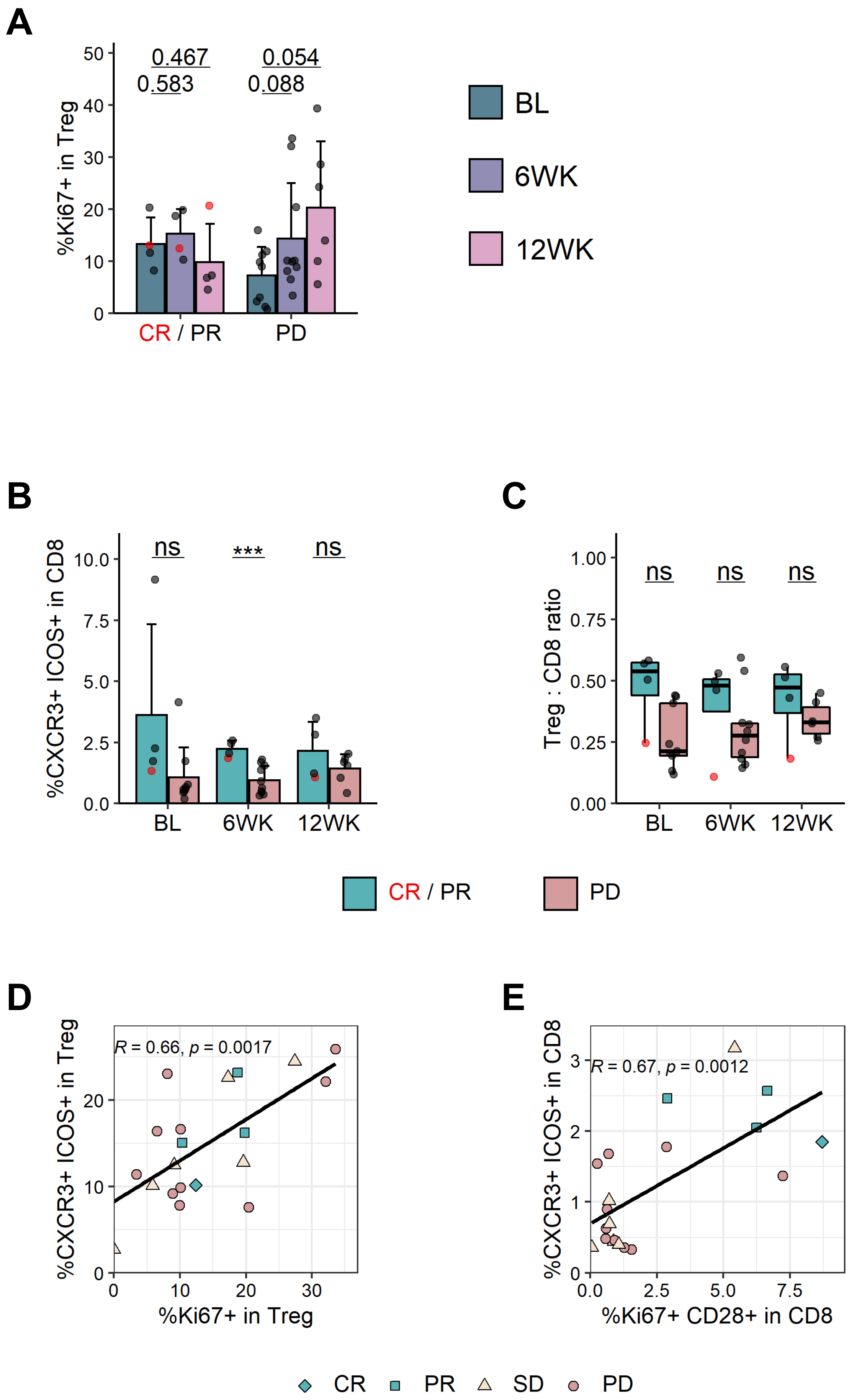

Suppl. Figure 4

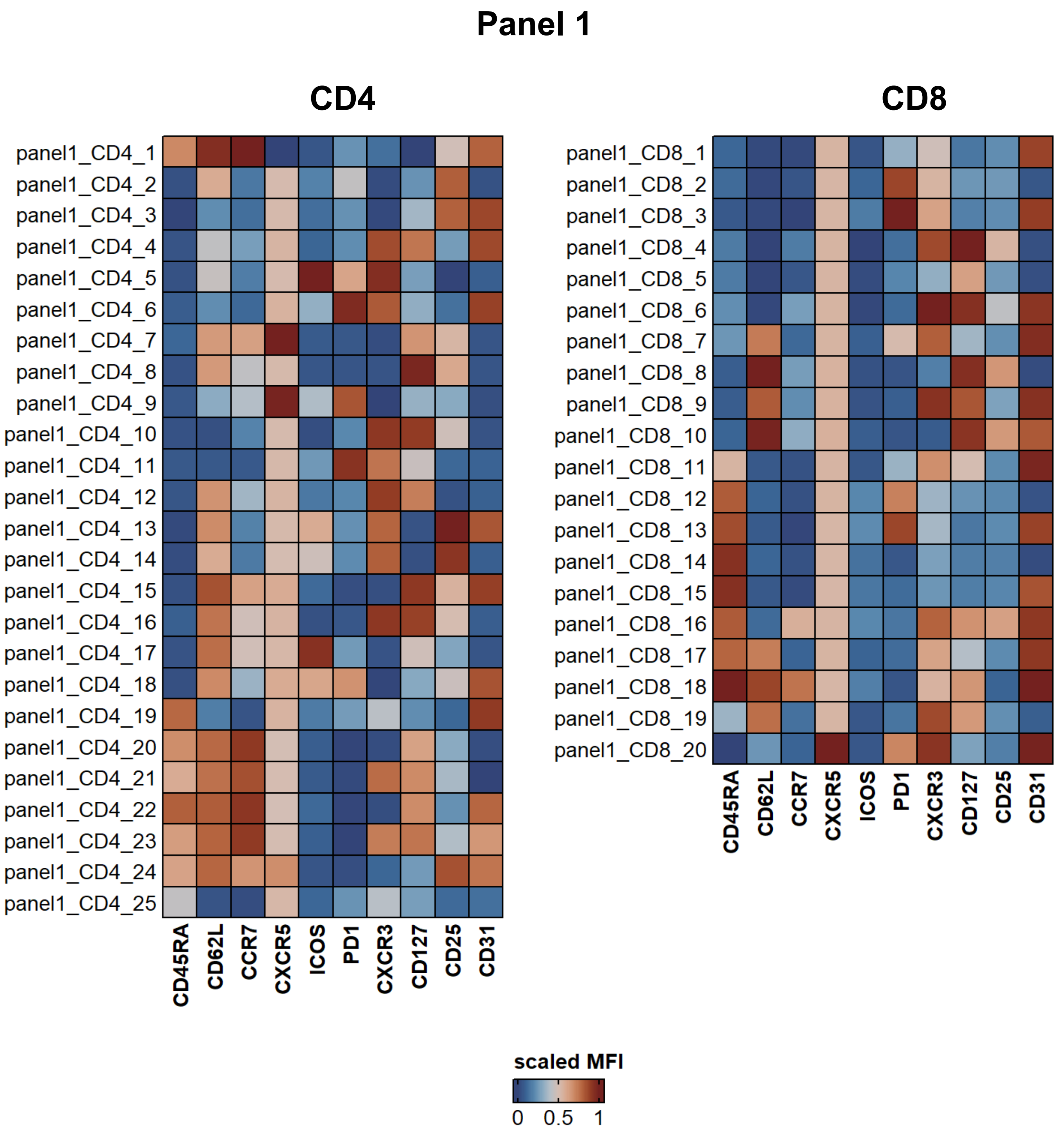

Suppl. Figure 5

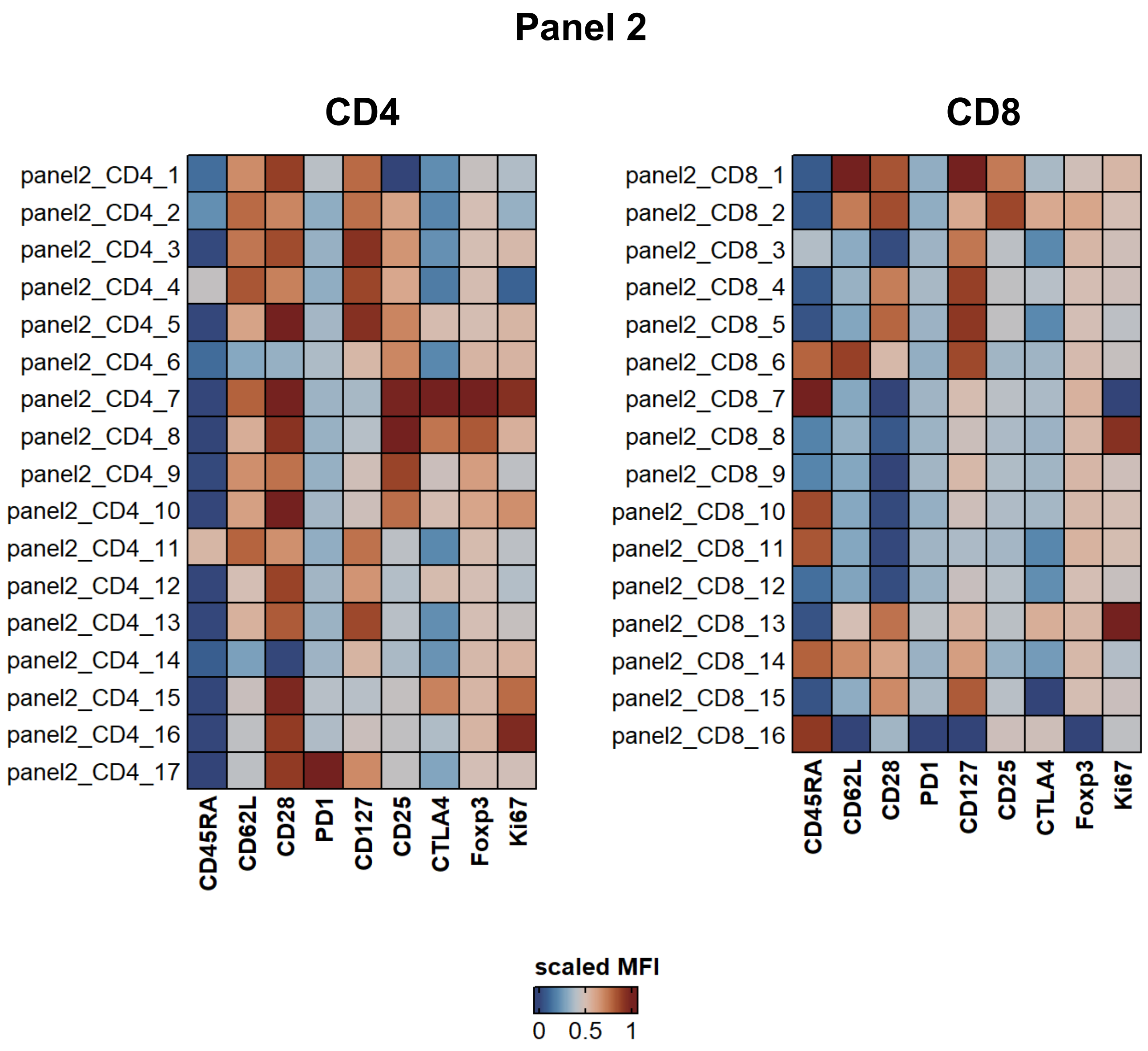

Suppl. Figure 5 (cont.)

Panel 3

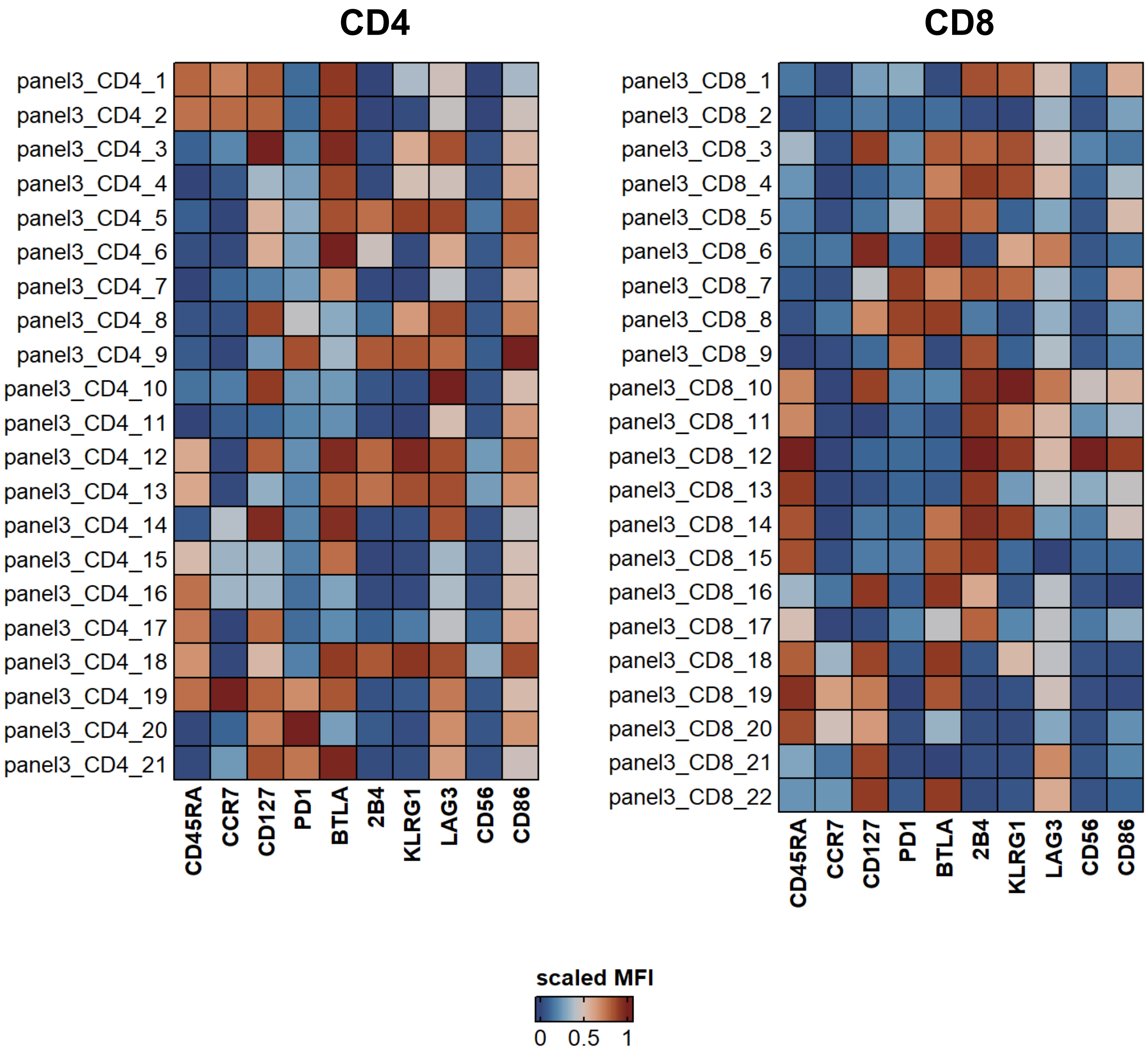

Suppl. Figure 5 (cont.)

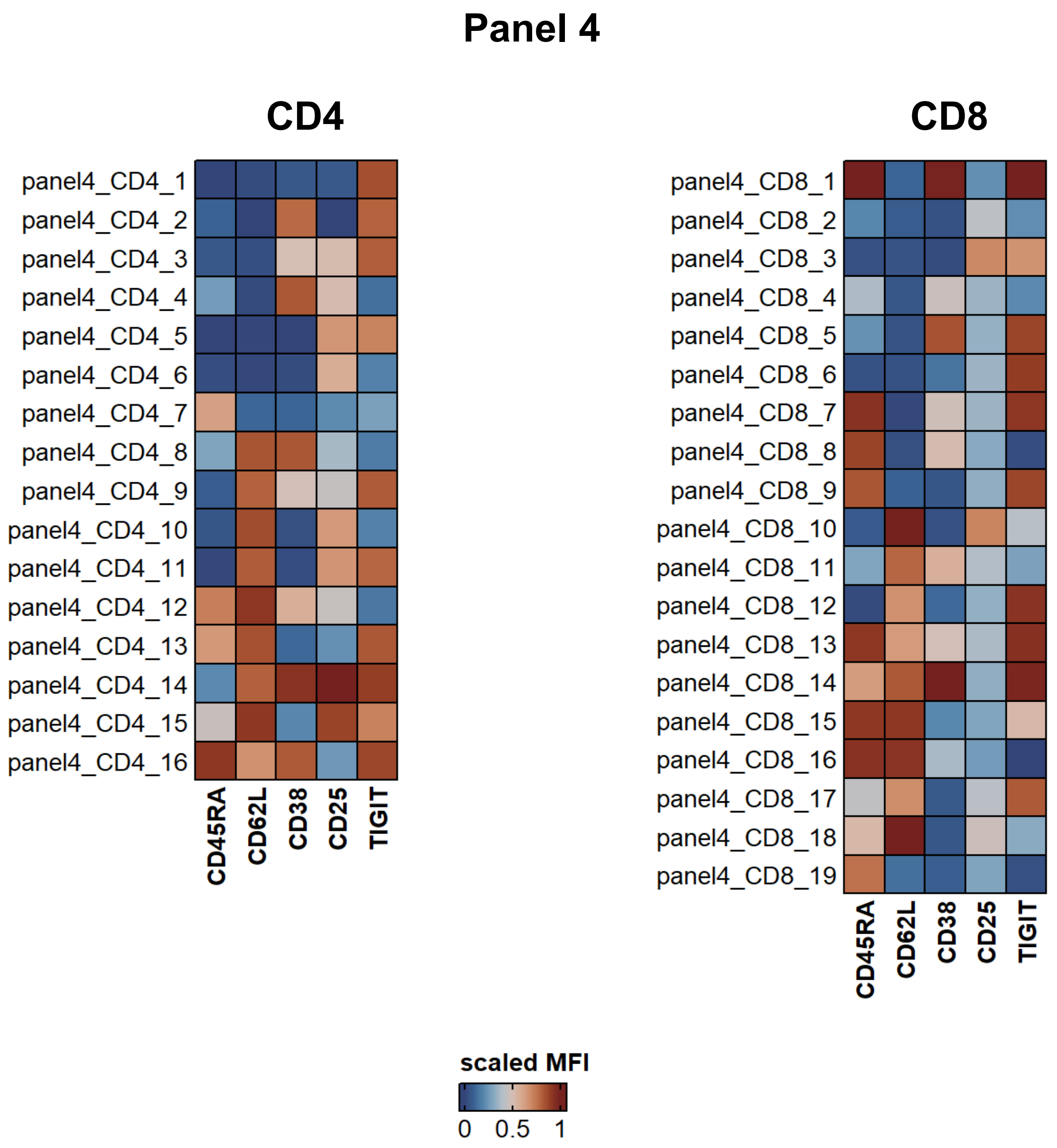

Suppl. Figure 5 (cont.)

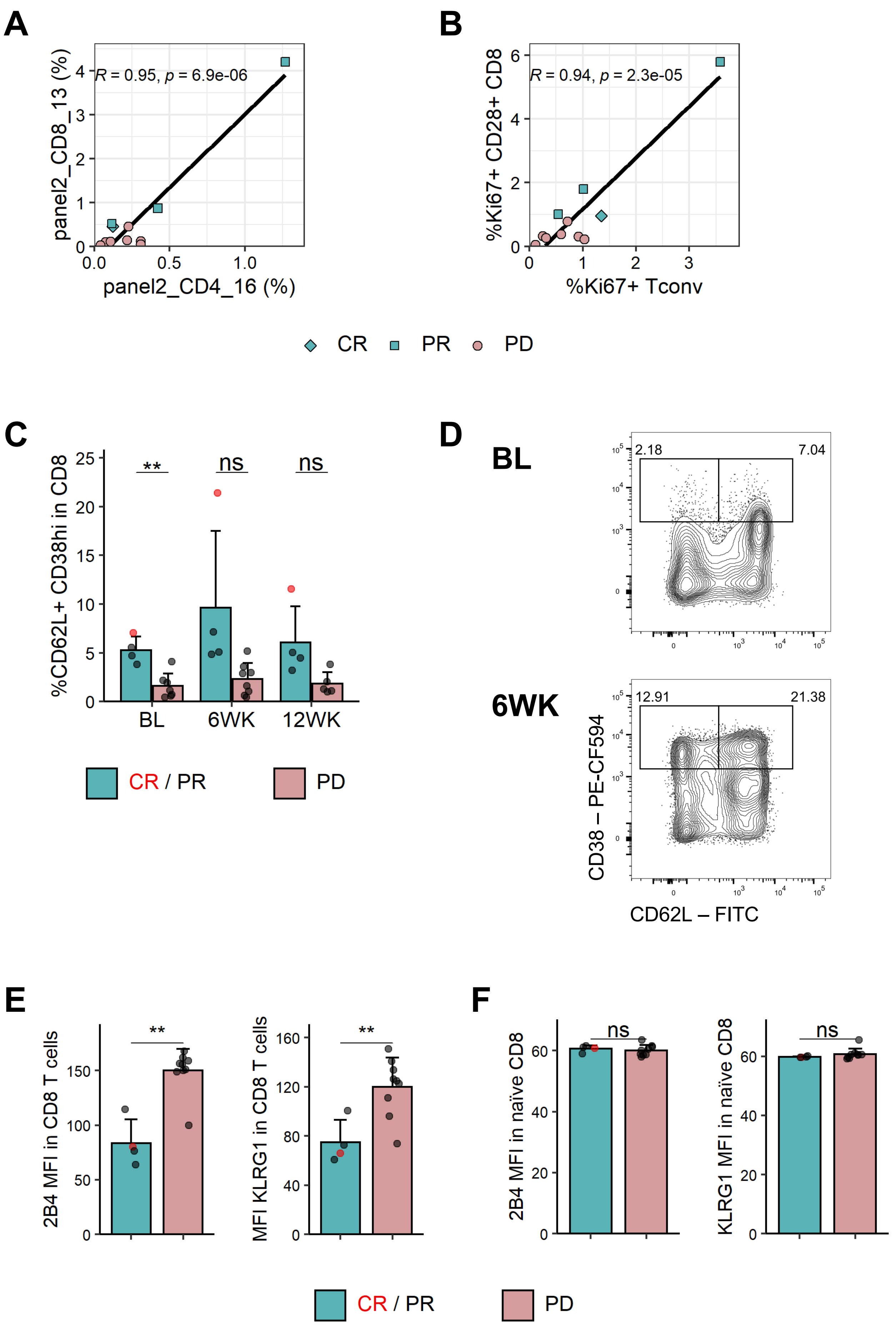

Suppl. Figure 6

and non-responders. CR, n = 1; PR, n = 3; PD, n = 7. (**B**) Top and bottom 15 FlowSOM clusters contributing to PC1 ordered by PC weight. (**C**) Heatmaps showing scaled MFI of indicated markers in CD8 T cell clusters shown in (**B**). (**D**) Heatmaps showing scaled MFIs of indicated markers in CD4 T cell clusters shown in (**B**). Arrows in (**C**) and (**D**) indicate directionality of associated PC1 weight. (**E**) CD28- frequency in CD8 T cells in baseline samples of responders and non-responders. (**F**) MFI of 2B4 (top) and KLRG1 (bottom) in CD8 T cells normalised to MFI in naïve (CD45RA+ CCR7+) CD8 T cells. Data shown is from baseline samples of responders and non-responders. (**E/F**) Shown are means + s.d.. CR/PR, n = 4; PD, n = 9. Two-tailed Student's t test; **, P < 0.01; *, P < 0.05.

Suppl. Figure S1 Frequencies of naïve and memory CD4 and CD8 T cells does not change following PD-1 blockade.

(**A**) Frequency of naïve (top left, CD45RA+ CD62L+), central memory (CM, top right, CD45RA- CD62L+), effector memory (EM, bottom left, CD45RA- CD62L-) and terminally differentiated (TEMRA, bottom right, CD45RA+ CD62L-) in CD4 T cells. (**B**) Frequency of naïve (top left, CD45RA+ CD62L+), central memory (CM, top right, CD45RA- CD62L+), effector memory (EM, bottom left, CD45RA- CD62L-) and terminally differentiated (TEMRA, bottom right, CD45RA+ CD62L-) in CD8 T cells. Shown are means + s.d.. BL, n = 19; 6WK, n = 20; 12WK, n = 16. Two-tailed Student's t test; ns, not significant.

Suppl. Figure S2 PD-1 detection in CD8 T cells following PD-1 blockade is the same in responders and non-responders.

(**A**) PD-1+ frequency in CD8 T cells. BL, n = 19; 6WK, n = 20; 12WK, n = 16. (**B**) PD-1+ frequency in CD4 T cells stratified by response. (**C**) PD-1+ frequency in CD8 T cells stratified by response. (**D**) (top) PD-1+ frequency in CD4 T cells in CR, PR, SD and PD patients. Points from same patient are connected by lines. Colour indicates PD-1+ frequency at baseline. (bottom) Ranking of PD-1 expression in CR, PR, SD and PD patients at baseline and the 6-week timepoint. For P23 no baseline bleed was available. (**B/C/D**) CR/PR, n = 4 (all time points); SD, n = 6 (all time points); PD, n = 9

(BL), n = 10 (6WK), n = 6 (12WK). Shown are means + s.d.. Two-tailed Student's t test; ****, P < 0.0001; *, P < 0.05; ns, not significant.

Suppl. Figure S3 Increase in proliferation in CD4 T cells does not distinguish responders and non-responders.

(**A**) Ki67+ frequency in CD4 T cells. (**B**) Representative flow cytometry plots showing Ki67 and CD28 expression in CD8 T cells in baseline, 6-week and 12-week bleeds of one PR and one PD patient. (**C**) Ki67+ frequency in CD8 T cells stratified by response. (**D**) Frequency of Ki67+ (left), Ki67+ CD28+ (middle) and Ki67+ CD28- (right) in CD4 T cells stratified by response. (**A**) BL, n = 19; 6WK, n = 20; 12WK, n = 16. (**C/D**) CR/PR, n = 4 (all time points); SD, n = 6 (all time points); PD, n = 9 (BL), n = 10 (6WK), n = 6 (12WK). Shown are means + s.d.. Two-tailed Student's t test; ns, not significant.

Suppl. Figure S4 CTLA-4+ Treg are transiently increased in non-responders following PD-1 blockade.

(**A**) Frequency of Treg (CD25+ CD127- Foxp3+ CTLA-4+) in CD4 T cells. (**B**) MFI of intracellular CTLA-4 in Treg (CD25+ CD127-) of non-responders. (**C**) Scaled histogram showing intracellular CTLA-4 expression in naïve T cells (filled) or Treg (open) from a non-responder at the indicated time points. (**D**) CXCR3+ ICOS+ frequency in Treg (CD25+ CD127-). (**E**) Ki67+ frequency in Treg. (**A/D/E**) Shown are means + s.d.. (**B**) Shown are box plots, with black horizontal line denoting median value, while box represents the IQRs (IQR, Q1–Q3 percentile) and whiskers show the minimum (Q1 − 1.5× IQR) and maximum (Q3 + 1.5× IQR) values. (**A/B/D/E**) CR/PR, n = 4 (all time points); SD, n = 6 (all time points); PD, n = 9 (BL), n = 10 (6WK), n = 6 (12WK). Two-tailed Student's t test; *, P < 0.05; ns, not significant.

Suppl. Figure S5 CXCR3 and ICOS expression correlates with proliferation of Treg and CD8 T cells.

(**A**) CXCR3+ ICOS+ frequency in CD8 T cells. (**B**) Ratio of CXCR3+ ICOS+ frequency in Treg to CXCR3+ ICOS+ frequency in CD8 T cells. (**C**) Ratio of Ki67+ frequency in

Treg to Ki67+ CD28+ frequency in CD8 T cells. (**D**) Ratio of Treg frequency (CD25+ CD127- Foxp3+ CTLA-4+) to CD8 T cell frequency. (**E**) Pearson correlation of Ki67+ frequency in Treg (CD25+ CD127- Foxp3+ CTLA-4+) to CXCR3+ ICOS+ frequency in Treg (CD25+ CD127-). (**F**) Pearson correlation of Ki67+ frequency to CXCR3+ ICOS+ frequency in CD8 T cells. (**A**) Shown are means + s.d.. (**B/C/D**) Shown are box plots, with black horizontal line denoting median value, while box represents the IQRs (IQR, Q1–Q3 percentile) and whiskers show the minimum (Q1 − 1.5× IQR) and maximum (Q3 + 1.5× IQR) values. (**A/B/C/D**) CR/PR, n = 4 (all time points); SD, n = 6 (all time points); PD, n = 9 (BL), n = 10 (6WK), n = 6 (12WK). Two-tailed Student's t test; ***, $P < 0.001$; *, $P < 0.05$; ns, not significant. (**E/F**) CR, n = 1; PR, n = 3; SD, n = 6; PD, n = 10. Pearson's R and associated p value are depicted on plots. Black line only for visualization purposes.

Suppl. Figure S6 Heatmaps of maker expression in FlowSOM clusters.

FlowSOM clustering was applied to CD4 and CD8 T cells stained with four distinct flow cytometry panels. Shown are heatmaps of scaled MFIs of indicated markers in CD4 (left) and CD8 (right) T cells.

Suppl. Figure S7 Clinical response following PD-1 blockade can be distinguished using baseline bleeds.

(**A**) CD28- frequency in CD8 T cells in baseline samples. (**B**) Pearson correlation of frequencies of FlowSOM clusters panel2_CD4_16 and panel2_CD8_13. (**C**) Pearson correlation of manually gated Ki67+ frequency in Tconv (non-Treg) and Ki67+ CD28+ frequency in CD8 T cells. (**D**) CD62L+ CD38hi frequency in CD8 T cells. (**E**) Flow cytometry plots showing CD62L and CD38 expression in CD8 T cells in baseline and 6-week bleeds of CR patient. (**A/D**) Shown are means + s.d.. CR/PR, n = 4 (all time points); SD, n = 6 (all time points); PD, n = 9 (BL), n = 10 (6WK), n = 6 (12WK). Two-tailed Student's t test; **, $P < 0.01$; *, $P < 0.05$; ns, not significant. (**B/C**) CR, n = 1; PR, n = 3; SD, n = 6; PD, n = 7. Pearson's R and associated p value are depicted on plots. Black line only for visualization purposes.

Suppl. Figure S8 CD8 T cells of non-responders have higher expression of 2B4 and KLRG1 at baseline

(**A**) MFI of 2B4 (left) and KLRG1 (right) in CD8 T cells in baseline samples. (**B**) Representative flow cytometry plots showing 2B4 and KLRG1 expression in CD8 T cells in baseline bleed of one PR patient and one PD patient. (**C**) MFI of 2B4 (left) and KLRG1 (right) in naïve (CD45RA+ CCR7+) CD8 T cells in baseline samples. (**D**) MFI of 2B4 (left) and KLRG1 (right) in CD8 T cells normalised to MFI in naïve (CD45RA+ CCR7+) CD8 T cells. Data shown is from baseline samples. (**A/C/D**) Shown are means + s.d.. CR/PR, n = 4; SD, n = 6; PD, n = 9. Two-tailed Student's t test; **, $P < 0.01$; ns, not significant.

Suppl. Figure S9 Gating strategies

Shown are representative gating strategies for relevant CD4 and CD8 T cell populations in indicated flow cytometry panels. For all panels, cells in first gate are live, singlet Lymphocytes. Grey background indicates same parent gate.

Patient ID	Gender	Performance status at registration	BRAF mutation	Pre-cycle 5 assessment	
				Radiological response	Adverse events
P02	Female	2	-	progression	Mild fatigue
P03	Male	0	Negative	progression	none
P04	Female	1	Negative	mixed response	Hypothyroidism
P05	Female	0	Negative	good response	-
P06	Female	0	Negative	borderline progression	-
P07	Male	0	Negative	progression	-
P10	Female	0	Positive (K601E)	mixed response	none
P11	Female	2	Negative	progression	none
P12	Male	0	Negative	partial response	none
P14	Male	0	Negative	stable disease	none
P15	Male	1	Negative	progression	-
P16	Female	0	Negative	progression	Chest infection
P17	Female	0	Negative	stable disease	unwell with cold, breathlessness
P19	Male	0	Negative	pseudoprogression	headaches affecting sleep, numbness in limbs
P20	Female	0	Negative	partial response	none
P21	Female	2	Negative	stable disease	none
P22	Female	0	Negative	progression	-
P23	Male	0	Positive	progression	-
P24	Female	1	Unknown	progression	-
P25	Female	2	Negative	mixed response	-

Panel 1

Target	Conjugate	Clone	Supplier
ICOS	biotin	ISA-3	Thermo Fisher Scientific
CD25	BV421	M-A251	BD Biosciences
CD3	BUV395	SK7	BD Biosciences
CD127	BV711	HIL-7R-M21	BD Biosciences
CXCR5	Alexa Fluor 488	RF8B2	BD Biosciences
CD45RA	PerCP-Cy5.5	HI100	Thermo Fisher Scientific
PD-1	PE	J105	Thermo Fisher Scientific
CD4	PE-Cy7	SK3	BD Biosciences
CD62L	Alexa Fluor 700	DREG-56	Biolegend
CCR7	BV605	G043H7	Biolegend
CXCR3	BV785	G025H7	Biolegend
CD31	PE-CF594	WM59	BD Biosciences

Panel 2

Target	Conjugate	Clone	Supplier
CD3	BUV395	SK7	BD Biosciences
CD25	BV421	M-A251	BD Biosciences
CD4	BV510	OKT4	Biolegend
CD28	BV650	CD28.2	Biolegend
PD-1	BV786	EH12.1	BD Biosciences
CD45RA	PerCP-Cy5.5	HI100	Thermo Fisher Scientific
CD8	PE-CF594	RPA-T8	BD Biosciences
CD127	PE-Cy7	IL-7R-M21	BD Biosciences
CD62L	Alexa Fluor 700	DREG-56	Biolegend

Panel 3

Target	Conjugate	Clone	Supplier
CD3	BUV395	SK7	BD Biosciences
PD-1	BV421	EH12.2H7	Biolegend
CD45RA	BV510	HI100	Biolegend
CCR7	BV650	G043H7	Biolegend
CD8	BV786	RPA-T8	BD Biosciences
LAG-3	FITC	3DS223H	Thermo Fisher Scientific
KLRG1	PerCP-eFluor 710	13F12F2	Thermo Fisher Scientific
BTLA	PE	J168-540	BD Biosciences
2B4	PE-Dazzle594	C1.7	Biolegend
CD127	PE-Cy7	IL-7R-M21	BD Biosciences
CD56	APC	B159	BD Biosciences
CD86	APC-R700	2331	BD Biosciences

Panel 4

Target	Conjugate	Clone	Supplier
CD3	BUV395	SK7	BD Biosciences
CD25	BV421	M-A251	BD Biosciences
CD62L	FITC	DREG-56	Biolegend
CD45RA	PerCP-Cy5.5	HI100	Thermo Fisher Scientific
CD38	PE-CF594	HIT2	BD Biosciences
CD4	PE-Cy7	SK3	BD Biosciences
TIGIT	APC	MBSA43	Thermo Fisher Scientific

Table legends

Suppl. Table S1 Patient characteristics

Shown are characteristics of patients enrolled in the PASIP study.

Suppl. Table S2 Flow cytometry surface panels

Shown are the four flow cytometry panels of antibodies that were used for surface staining of PBMCs in this study.
