# Supplementary material for: Stratification of PD-1 blockade response in melanoma using pre- and post-treatment immunophenotyping of peripheral blood
Source: Immunother Adv. 2023 Jan 6;3(1):ltad001. doi: 10.1093/immadv/ltad001 (PMC9929715; doi:10.1093/immadv/ltad001)
Supplement: Supplementary file 1 [file ltad001_suppl_supplementary_figures.pdf]

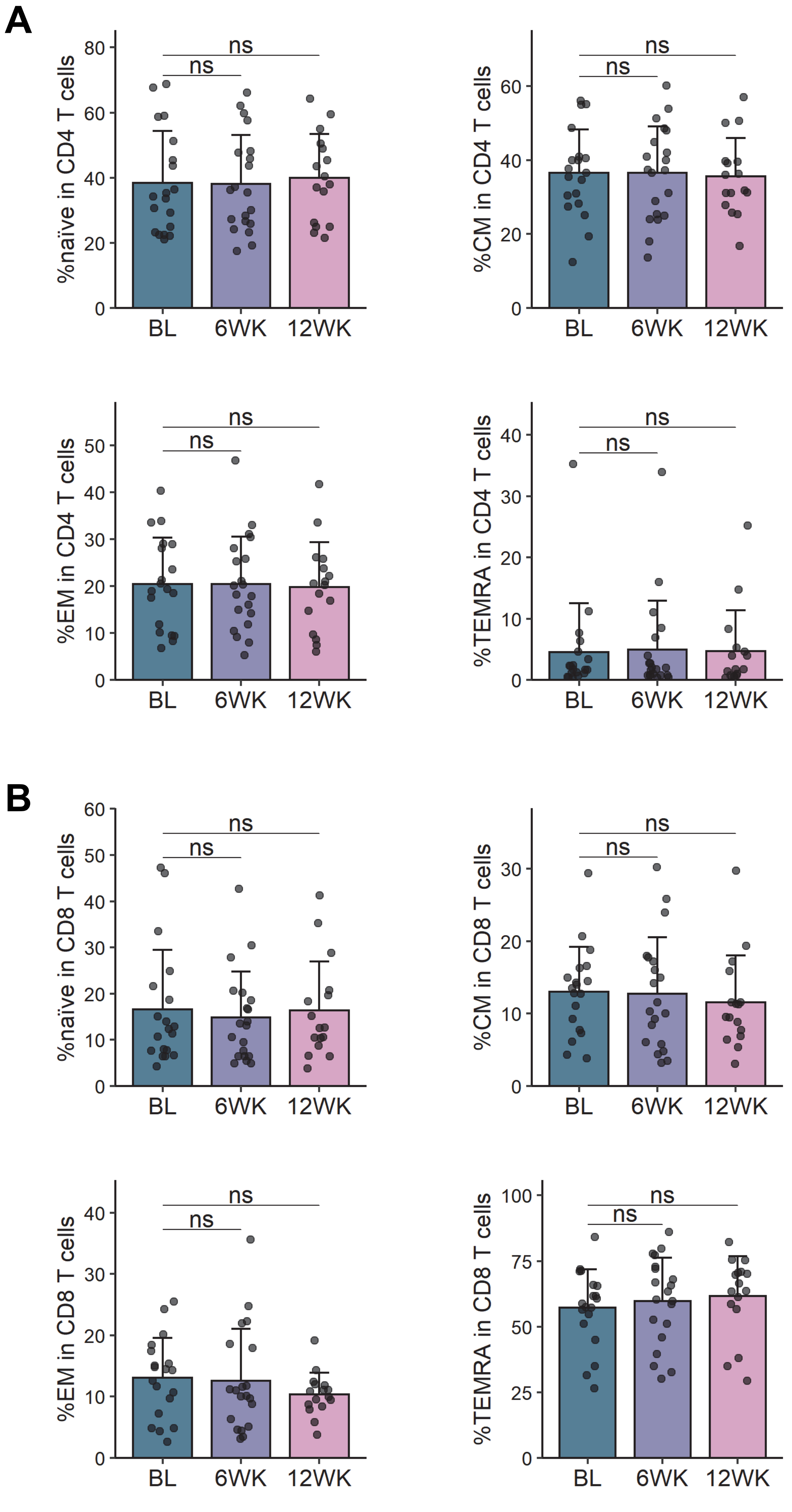

Suppl. Figure 1

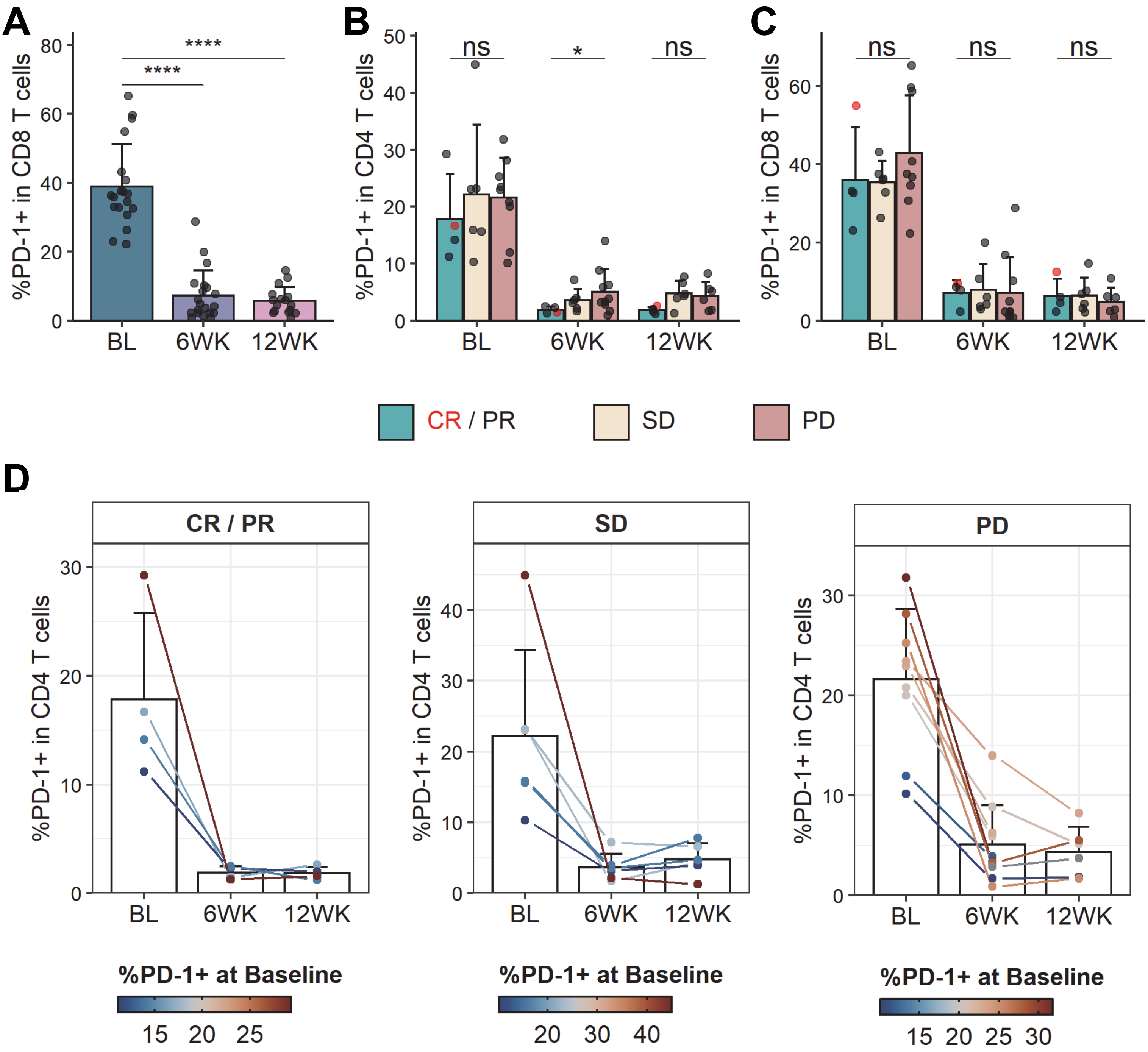

Patient	P21	P19	P25	P22	P24	P16	P06	P04	P15	P11	P07	P05	P17	P14	P20	P02	P12	P10	P03	P23
Response	SD	PD	PR	PD	PD	PD	SD	SD	PD	PD	PD	CR	SD	SD	PR	PD	PR	SD	PD	PD
Rank BL	1	2	3	4	5	6	7	8	9	10	11	12	13	14	15	16	17	18	19	-
Rank 6WK	15	9	19	10	20	1	16	3	4	2	5	18	8	6	13	7	14	11	17	12

Suppl. Figure 2

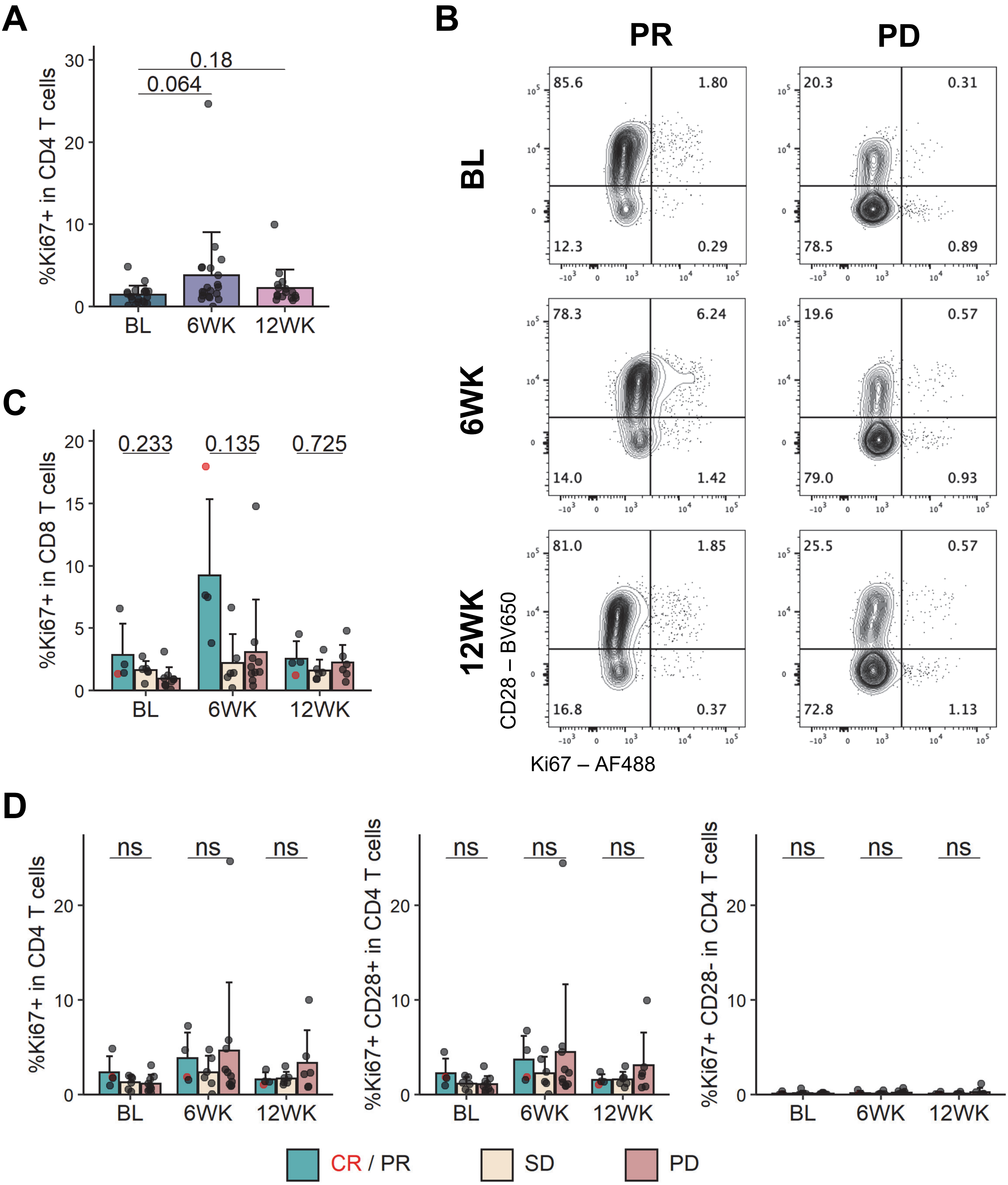

Suppl. Figure 3

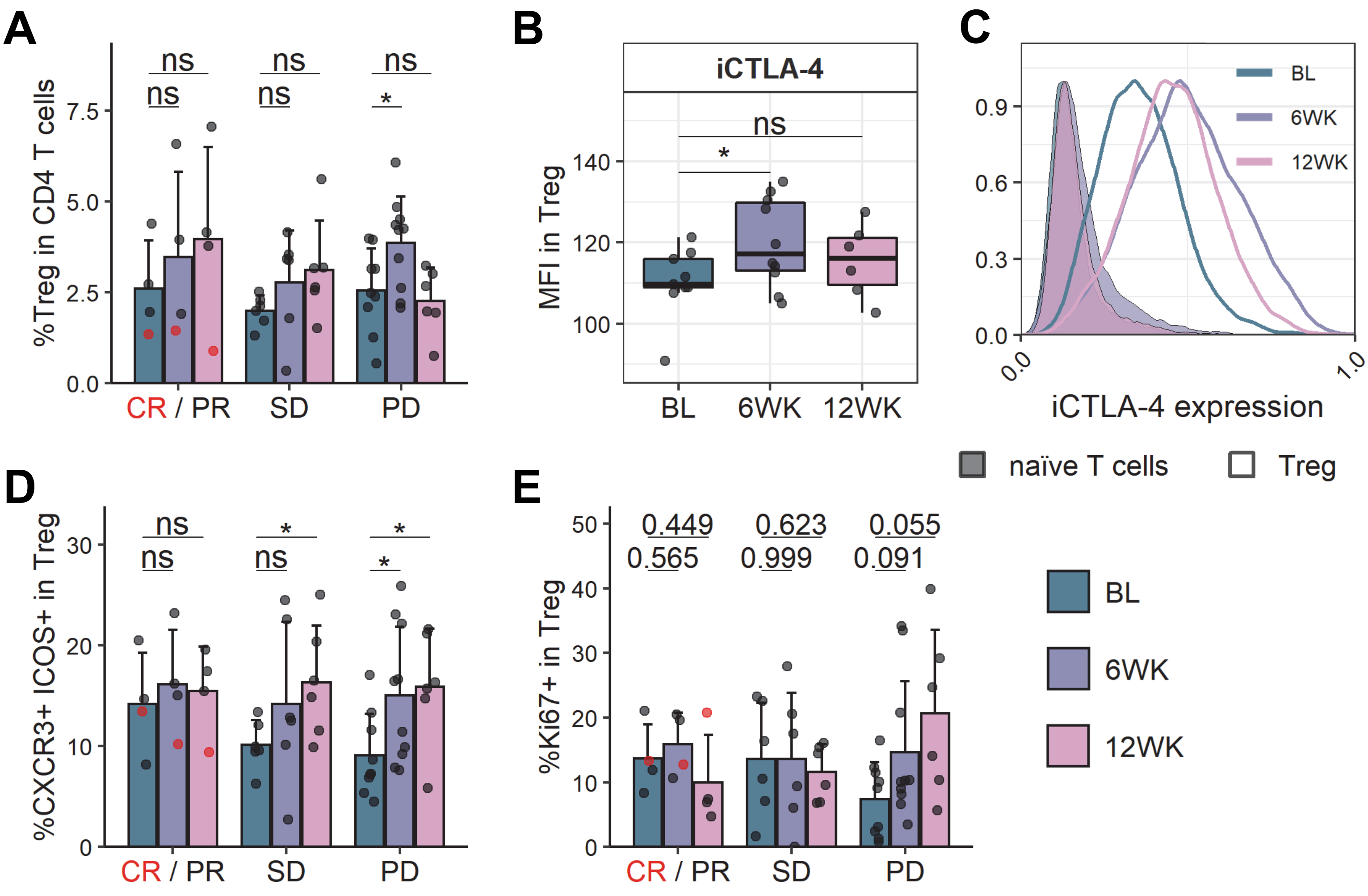

Suppl. Figure 4

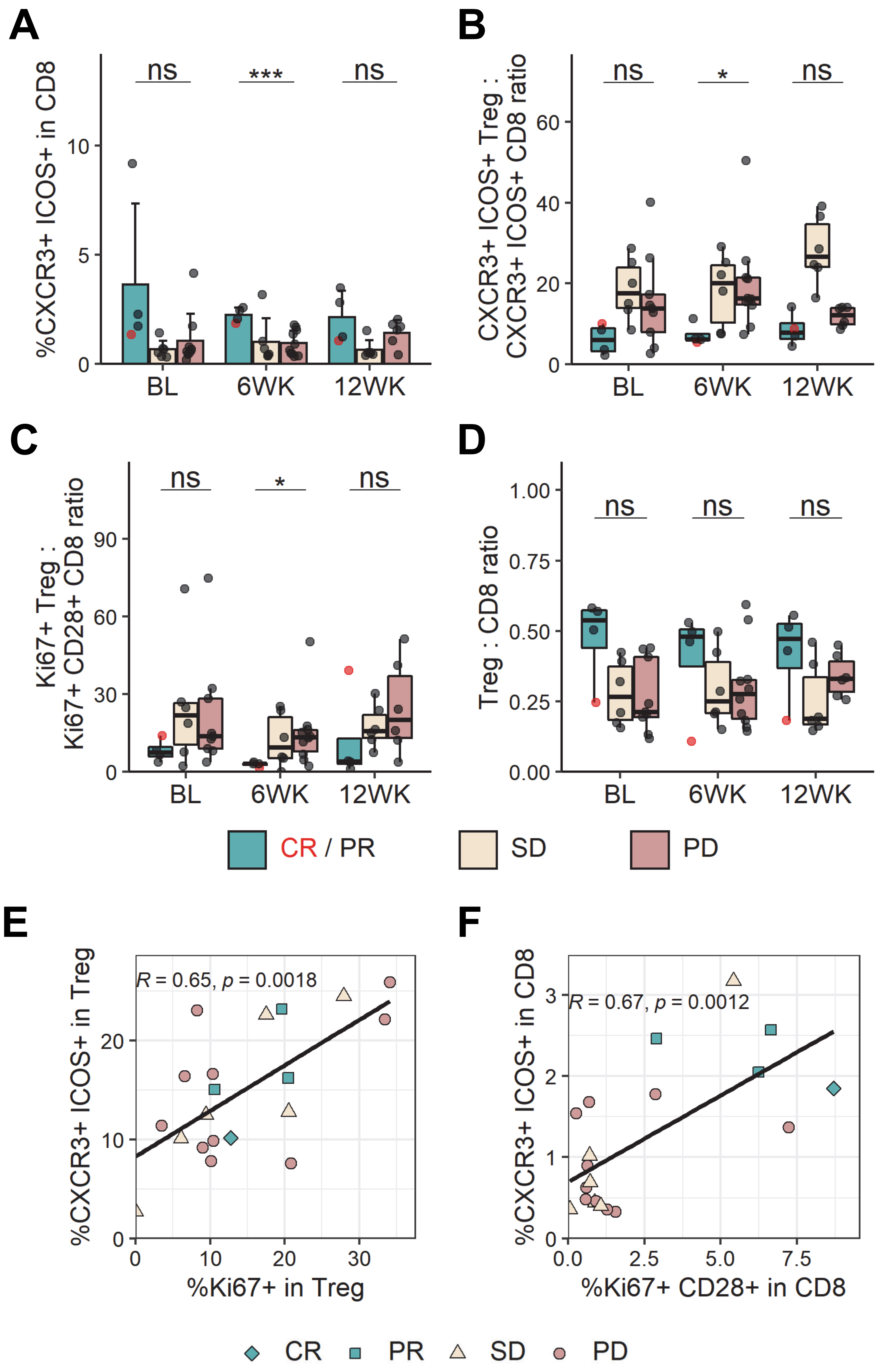

Suppl. Figure 5

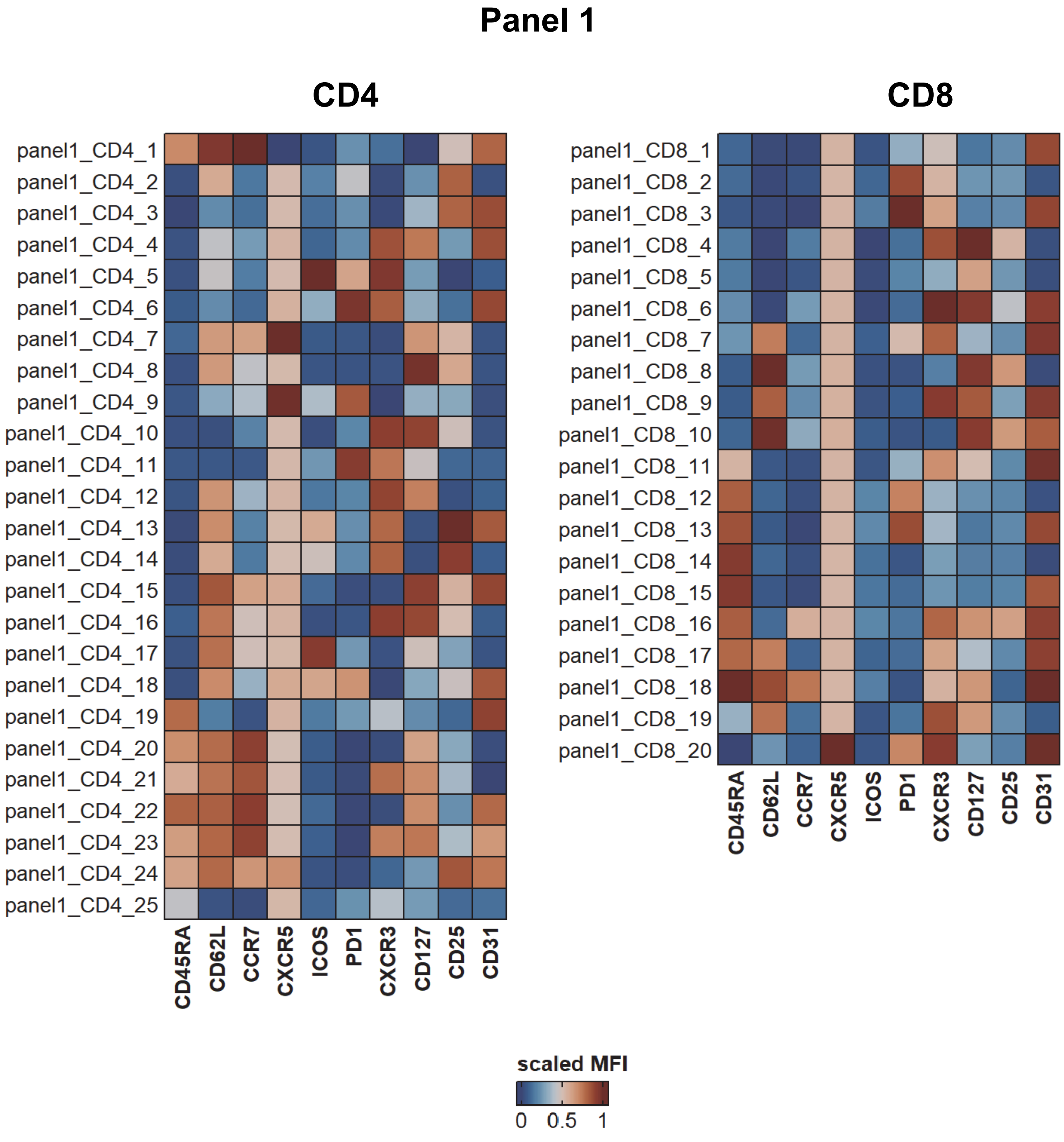

Suppl. Figure 6

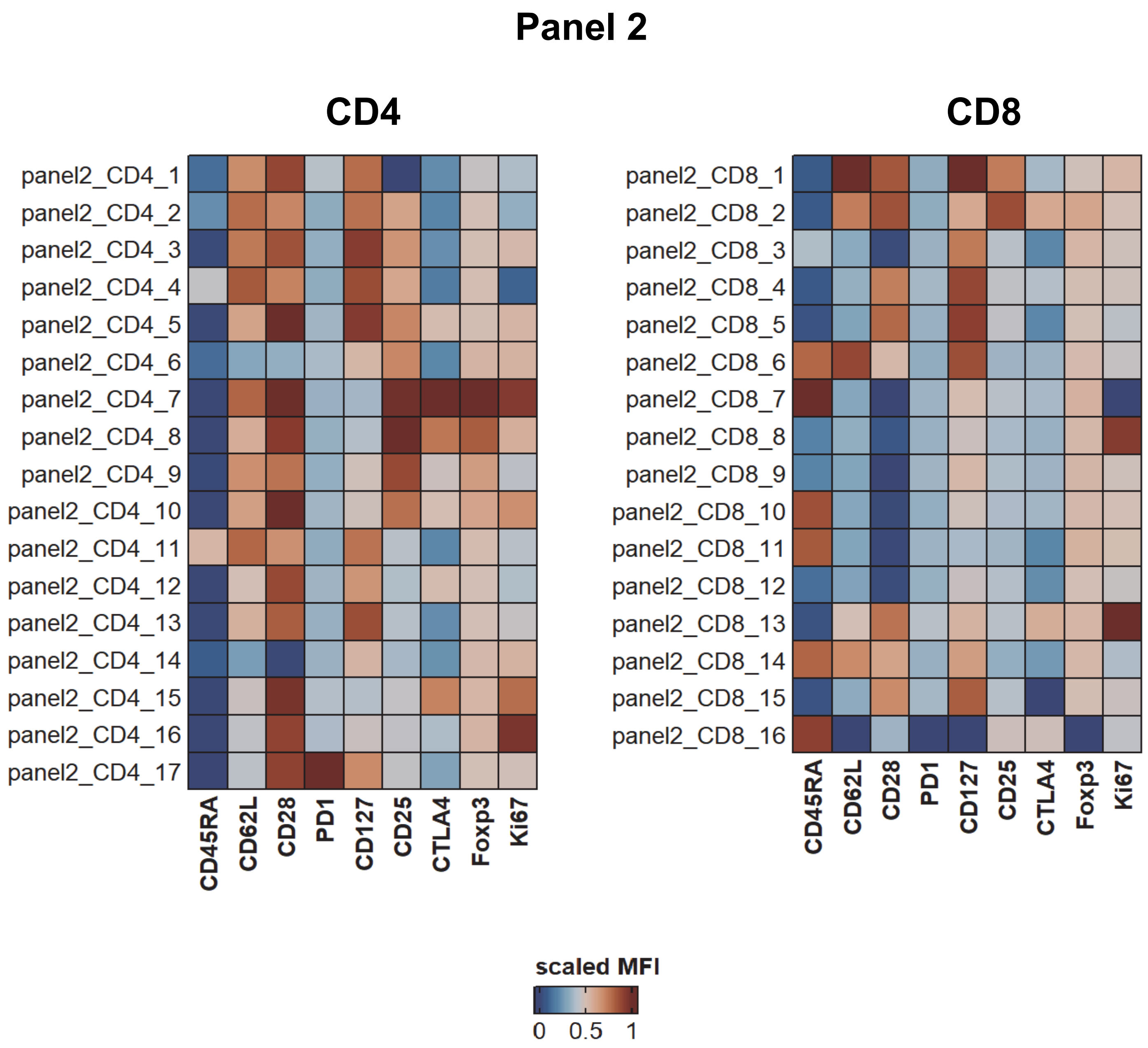

Suppl. Figure 6 (cont.)

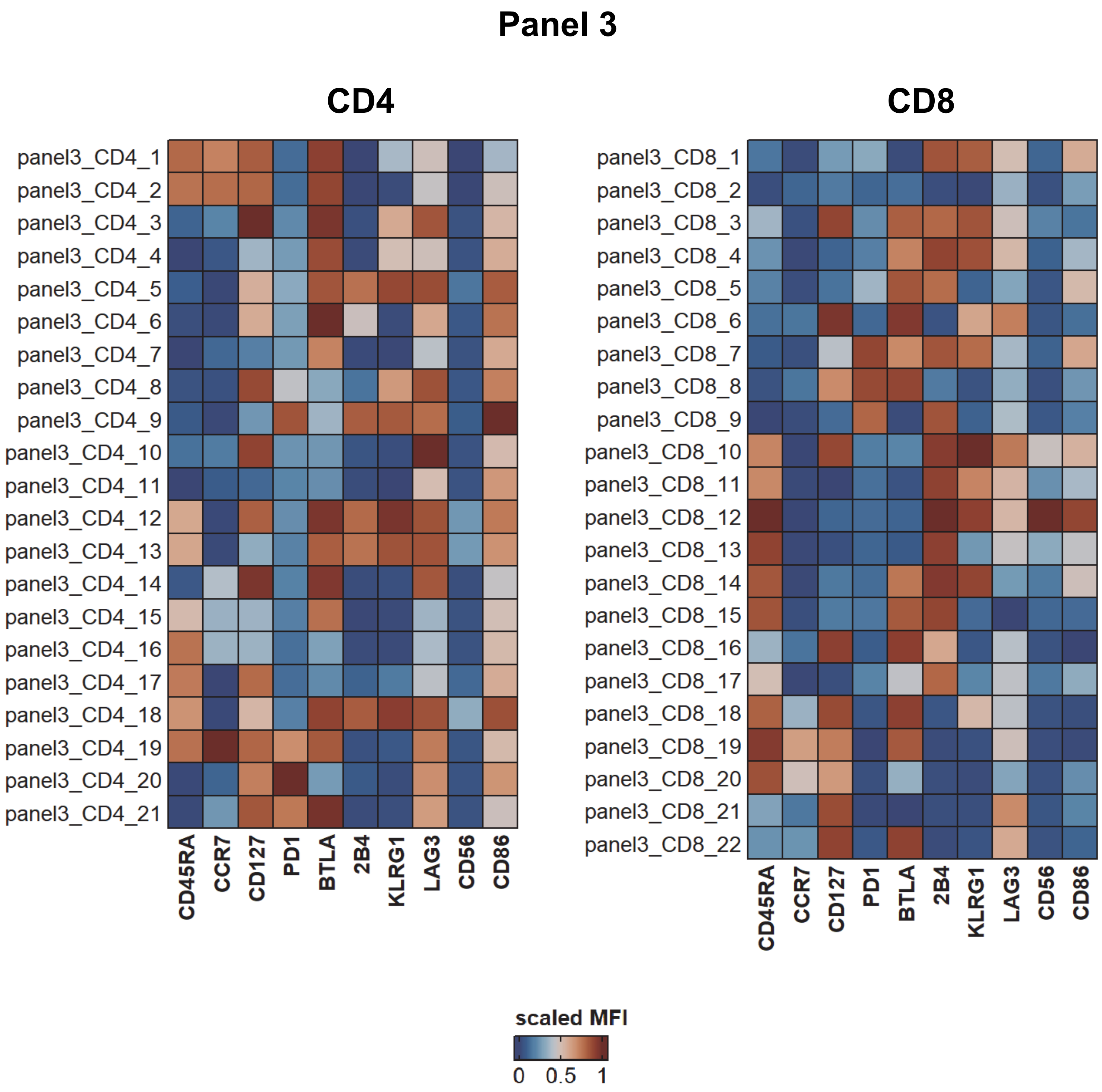

Suppl. Figure 6 (cont.)

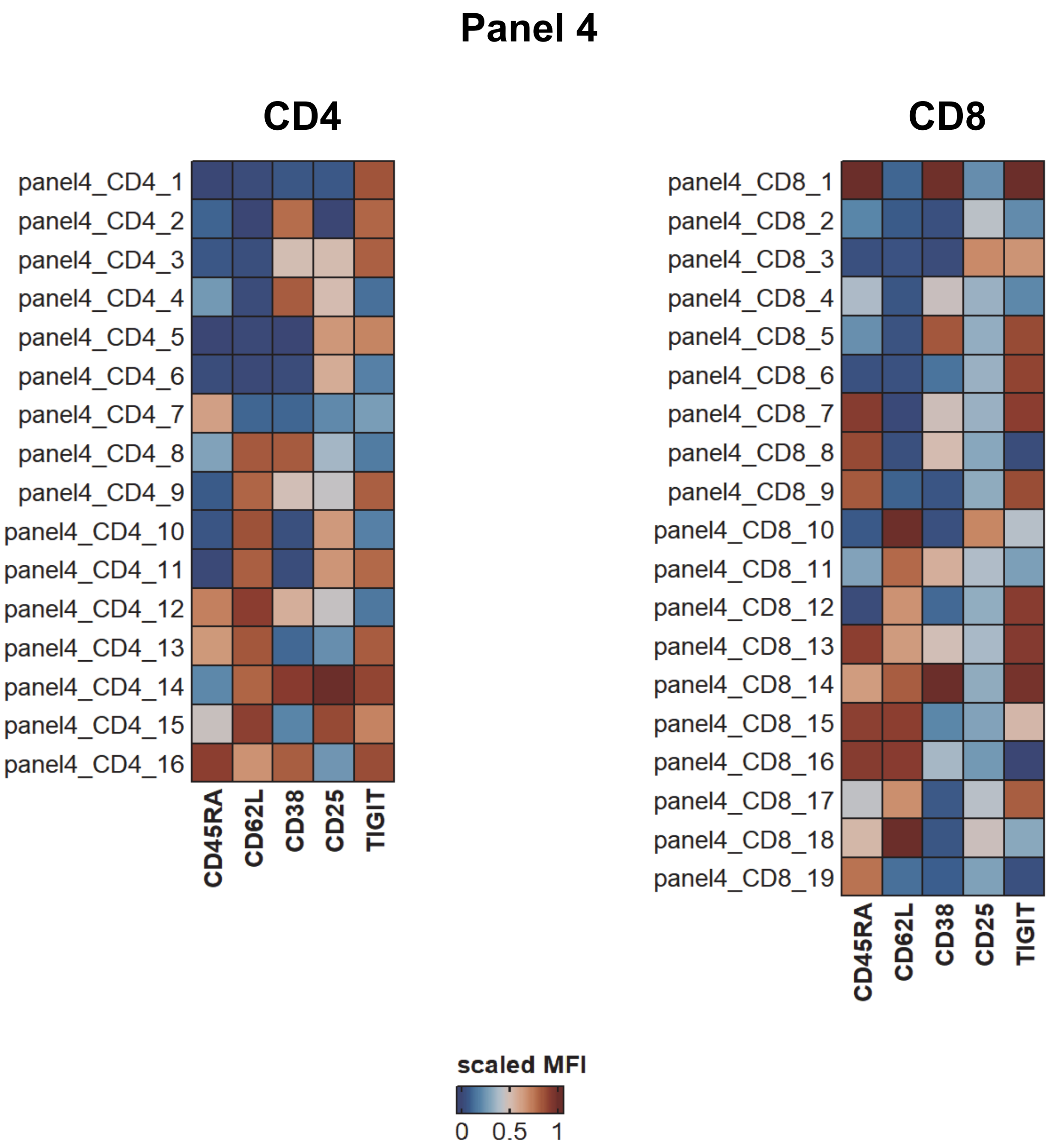

Suppl. Figure 6 (cont.)

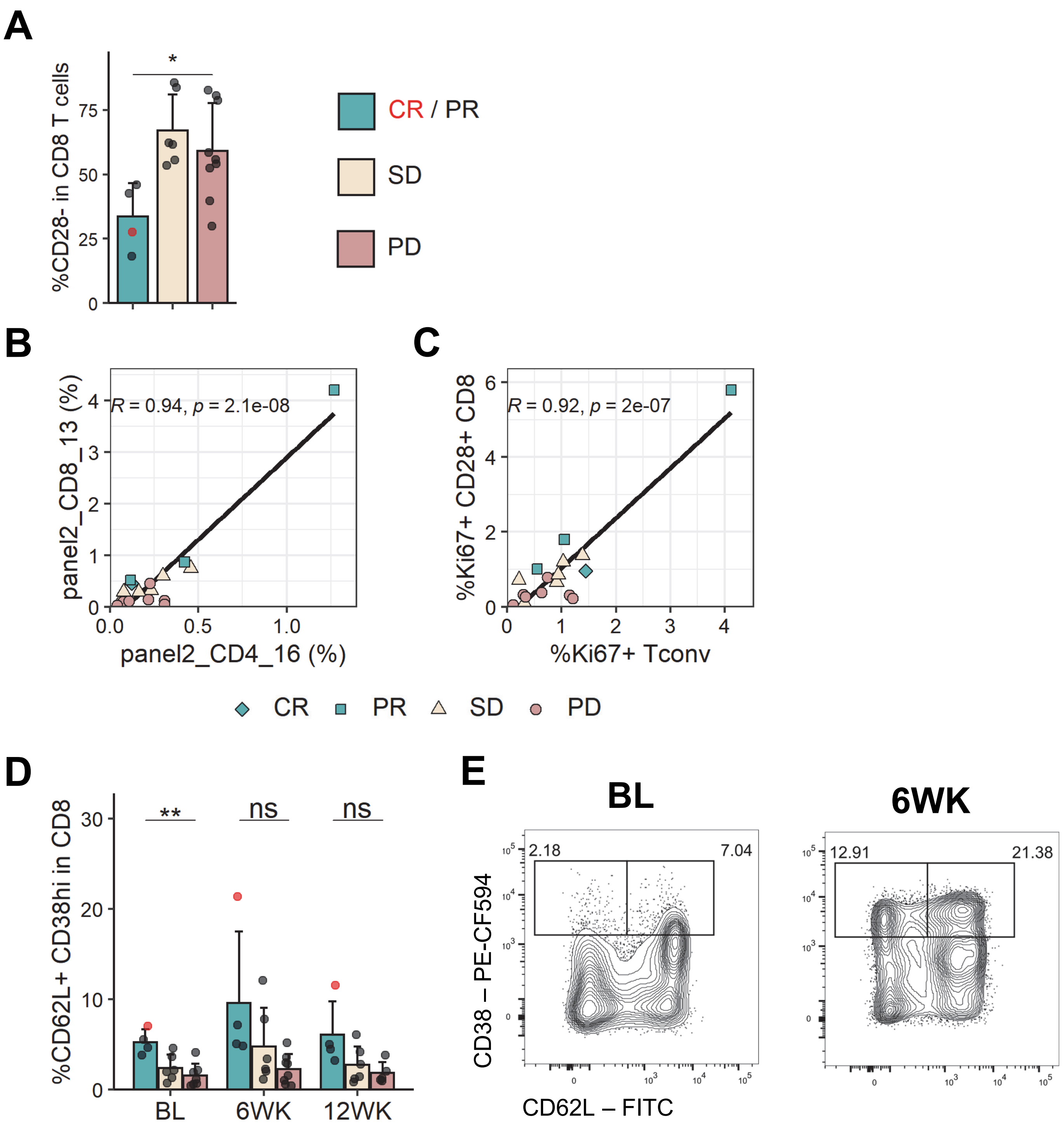

Suppl. Figure 7

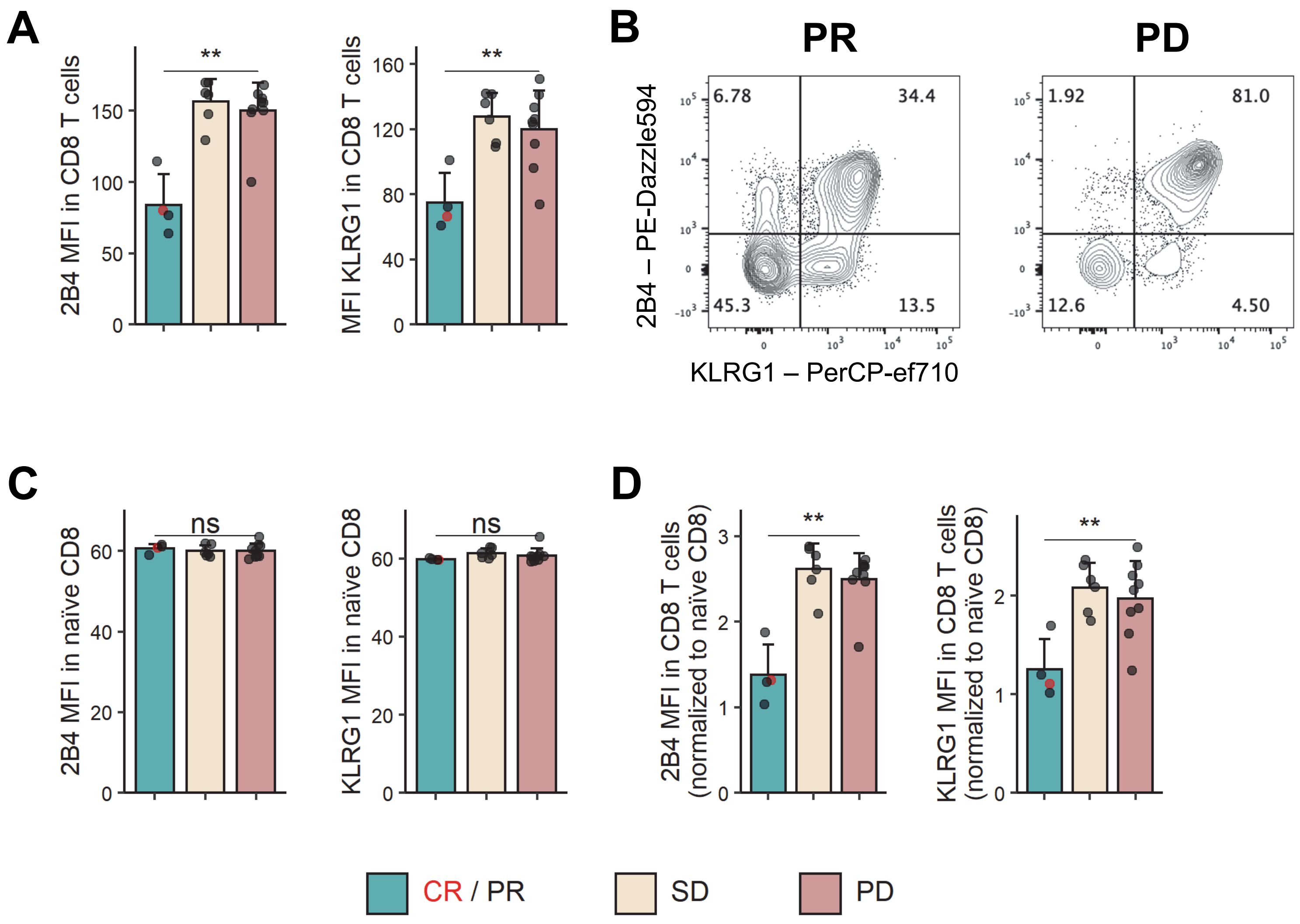

Suppl. Figure 8

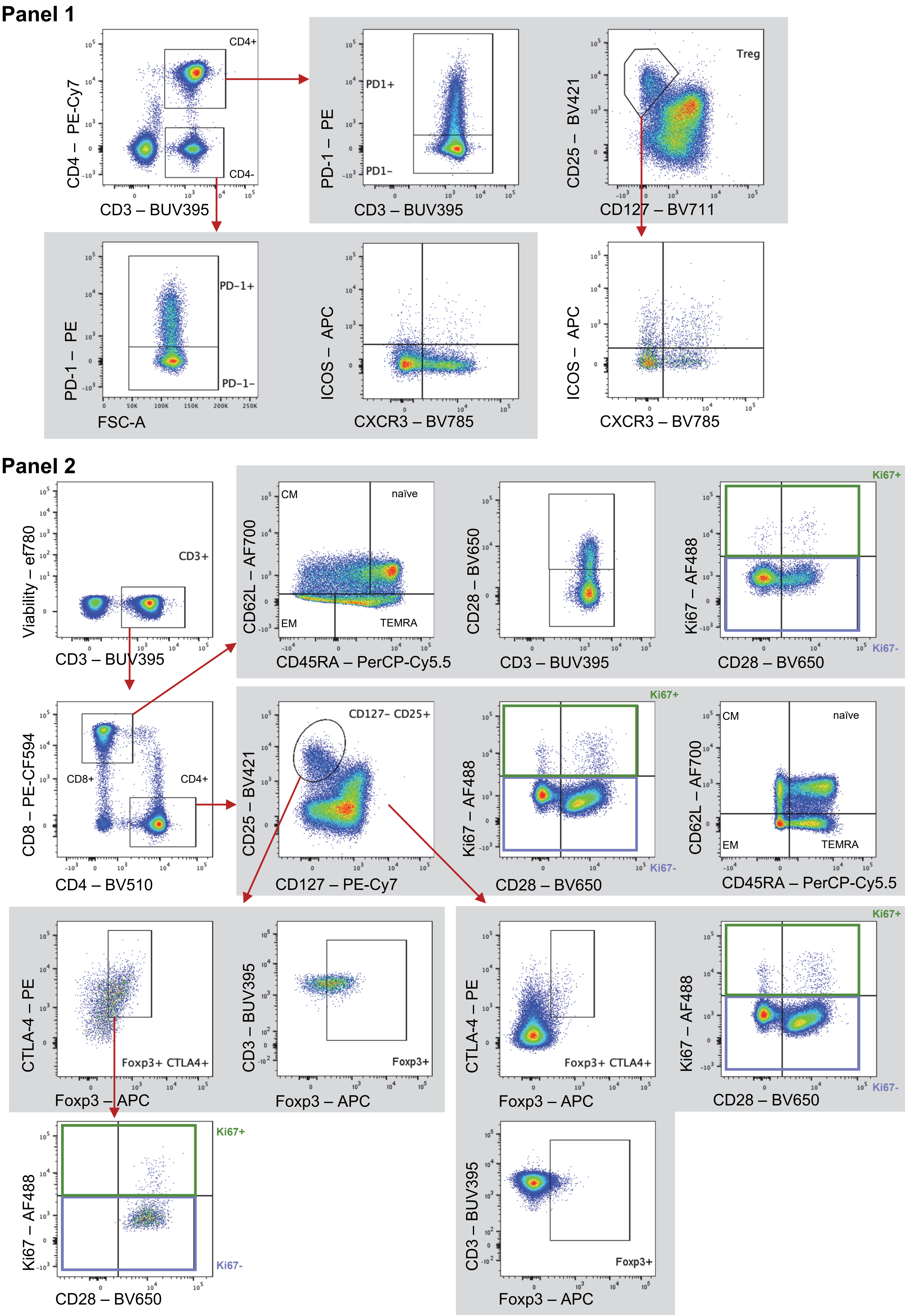

Suppl. Figure 9

Panel 3

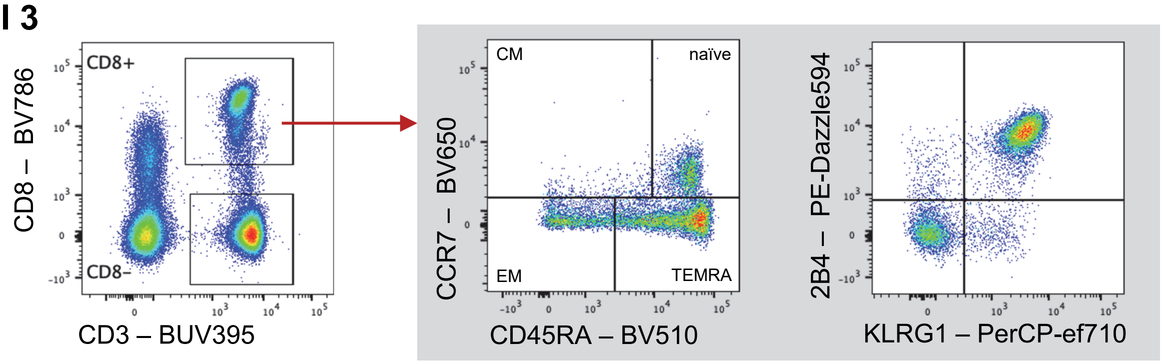

Panel 4

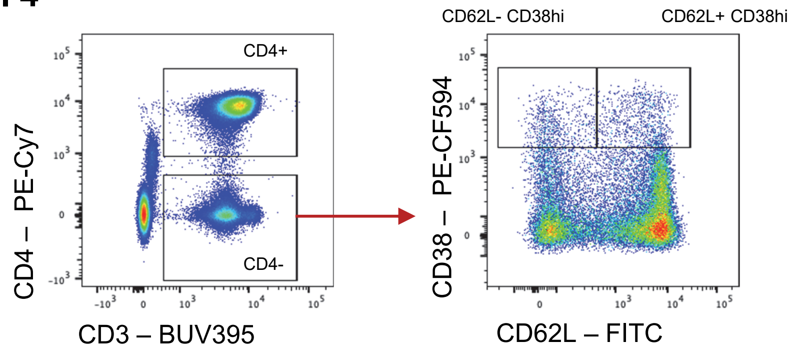

Suppl. Figure 9 (cont.)
