# Supplementary material for: Stratification of PD-1 blockade response in melanoma using pre- and post-treatment immunophenotyping of peripheral blood
Source: Immunother Adv. 2023 Jan 6;3(1):ltad001. doi: 10.1093/immadv/ltad001 (PMC9929715; doi:10.1093/immadv/ltad001)
Supplement: Supplementary file 2 [file ltad001_suppl_supplementary_table_s1.pdf]

Patient ID	Gender	Performance status at registration	BRAF mutation	Pre-cycle 5 assessment	
				Radiological response	Adverse events
P02	Female	2	-	progression	Mild fatigue
P03	Male	0	Negative	progression	none
P04	Female	1	Negative	mixed response	Hypothyroidism
P05	Female	0	Negative	good response	-
P06	Female	0	Negative	borderline progression	-
P07	Male	0	Negative	progression	-
P10	Female	0	Positive (K601E)	mixed response	none
P11	Female	2	Negative	progression	none
P12	Male	0	Negative	partial response	none
P14	Male	0	Negative	stable disease	none
P15	Male	1	Negative	progression	-
P16	Female	0	Negative	progression	Chest infection
P17	Female	0	Negative	stable disease	unwell with cold, breathlessness
P19	Male	0	Negative	pseudoprogression	headaches affecting sleep, numbness in limbs
P20	Female	0	Negative	partial response	none
P21	Female	2	Negative	stable disease	none
P22	Female	0	Negative	progression	-
P23	Male	0	Positive	progression	-
P24	Female	1	Unknown	progression	-
P25	Female	2	Negative	mixed response	-
