# Supplementary material for: Stratification of PD-1 blockade response in melanoma using pre- and post-treatment immunophenotyping of peripheral blood
Source: Immunother Adv. 2023 Jan 6;3(1):ltad001. doi: 10.1093/immadv/ltad001 (PMC9929715; doi:10.1093/immadv/ltad001)
Supplement: Supplementary file 3 [file ltad001_suppl_supplementary_table_s2.pdf]

Panel 1

Target	Conjugate	Clone	Supplier
ICOS	biotin	ISA-3	Thermo Fisher Scientific
CD25	BV421	M-A251	BD Biosciences
CD3	BUV395	SK7	BD Biosciences
CD127	BV711	HIL-7R-M21	BD Biosciences
CXCR5	Alexa Fluor 488	RF8B2	BD Biosciences
CD45RA	PerCP-Cy5.5	HI100	Thermo Fisher Scientific
PD-1	PE	J105	Thermo Fisher Scientific
CD4	PE-Cy7	SK3	BD Biosciences
CD62L	Alexa Fluor 700	DREG-56	Biolegend
CCR7	BV605	G043H7	Biolegend
CXCR3	BV785	G025H7	Biolegend
CD31	PE-CF594	WM59	BD Biosciences

Panel 2

Target	Conjugate	Clone	Supplier
CD3	BUV395	SK7	BD Biosciences
CD25	BV421	M-A251	BD Biosciences
CD4	BV510	OKT4	Biolegend
CD28	BV650	CD28.2	Biolegend
PD-1	BV786	EH12.1	BD Biosciences
CD45RA	PerCP-Cy5.5	HI100	Thermo Fisher Scientific
CD8	PE-CF594	RPA-T8	BD Biosciences
CD127	PE-Cy7	IL-7R-M21	BD Biosciences
CD62L	Alexa Fluor 700	DREG-56	Biolegend

Panel 3

Target	Conjugate	Clone	Supplier
CD3	BUV395	SK7	BD Biosciences
PD-1	BV421	EH12.2H7	Biolegend
CD45RA	BV510	HI100	Biolegend
CCR7	BV650	G043H7	Biolegend
CD8	BV786	RPA-T8	BD Biosciences
LAG-3	FITC	3DS223H	Thermo Fisher Scientific
KLRG1	PerCP-eFluor 710	13F12F2	Thermo Fisher Scientific
BTLA	PE	J168-540	BD Biosciences
2B4	PE-Dazzle594	C1.7	Biolegend
CD127	PE-Cy7	IL-7R-M21	BD Biosciences
CD56	APC	B159	BD Biosciences
CD86	APC-R700	2331	BD Biosciences

Panel 4

Target	Conjugate	Clone	Supplier
CD3	BUV395	SK7	BD Biosciences
CD25	BV421	M-A251	BD Biosciences
CD62L	FITC	DREG-56	Biolegend
CD45RA	PerCP-Cy5.5	HI100	Thermo Fisher Scientific
CD38	PE-CF594	HIT2	BD Biosciences
CD4	PE-Cy7	SK3	BD Biosciences
TIGIT	APC	MBSA43	Thermo Fisher Scientific
